# Climate‐Driven Body Size Changes in Birds and Mammals Reveal Environmental Tolerance Limits

**DOI:** 10.1111/gcb.70241

**Published:** 2025-05-09

**Authors:** Matthew J. Watson, Jeremy T. Kerr

**Affiliations:** ^1^ Department of Biology University of Ottawa Ottawa Ontario Canada

**Keywords:** adaptation, aridity niche, bird, body size, climate change, mammal, terrestrial vertebrate, thermal niche

## Abstract

Climate change contributes to widespread shifts in body size across taxa which can impact population and community dynamics. However, the reasons for variability in the direction and intensity of responses remain uncertain. Smaller body size improves thermoregulatory efficiency but can increase dehydration risk. Changes in species' body size is likely to balance the tradeoffs of thermoregulation and osmotic balance when responding to shifts in thermal and aridity regimes associated with climate change. Using 119,183 bird and 183,087 mammal body mass, and 15,562 bird and 239,600 mammal body length records, along with species' thermal and aridity limits based on their range geographies, we tested for associations between body size and climatic conditions. We also assessed the impacts of human land use extent and interactions with species thermal environments. We found that smaller body mass measurements across taxa are associated with conditions closer to species' upper thermal (hot) and lower aridity (dry) tolerance limits. Agricultural land use extent was found to be positively associated with body mass measurements for both bird and mammal species. Shorter body lengths were observed for both birds and mammals the closer species were to their upper thermal limits. Further we found that thermal and aridity conditions interacted resulting in stronger negative associations between body mass and hotter temperatures the closer species were to their dry tolerance limits. Our results are consistent with predictions that differences in body size within bird and mammal species are driven by thermoregulatory pressures associated with thermal and aridity regimes. While species' range geographies and phenology are widely known to respond to anthropogenic climate change, the shifts in species' body sizes detected here are a third biotic response that exerts similarly profound ecological, evolutionary, and conservation effects.

## Graphical abstract

Within bird and mammal species, individuals with smaller body mass and shorter body length measurements occupy habitats in closer proximity to warm niche limits. Additionally, habitats closer to species dry niche limits were associated with smaller body sizes in bird and mammal species. Aridity and thermal conditions were also found to interact, resulting in a strengthened association of smaller body sizes with habitats closer to species warm niche limits.

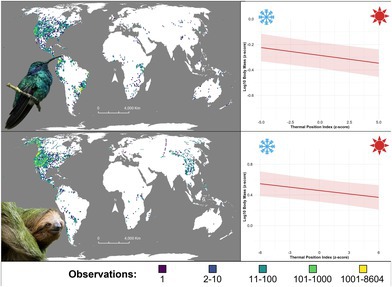

## 1 Introduction

Climate change contributes significantly to global extinction rates (Ehrlich et al. 2010), threatening one in six species (Urban 2015). Anthropogenic climate change is altering environmental conditions at a much faster rate than historic natural climate changes, while also increasing the frequency and intensity of heatwaves and droughts (Boyles et al. 2011; IPCC 2022). However, rates and effects of climate change show marked spatial variation (Harris et al. 2018; IPCC 2022; Urban 2015). The imposition of extreme temperatures and precipitation regimes associated with climate change exerts strong selective pressures on species, reducing reproductive output and increasing mortality rates, population‐level extinction rates (Conradie et al. 2019; Hansen 2009; Van de Ven et al. 2020), and species‐level extinction risks (Ehrlich et al. 2010; Pimm et al. 2014). Responses to climate change through geographical range shifts and phenological shifts are widely observed (Boyles et al. 2011; McCain and King 2014; Riddell et al. 2021), and appear vital for species' persistence. Yet, many key life history processes depend on organismal body size, which reflects life‐long interactions with the organism's environment.

In endothermic vertebrates, body size has been observed to affect species' thermoregulatory capacities, and varies broadly with temperature (Weathers 1981; McCain and King 2014). Smaller body sizes decrease an organism's volume‐to‐surface‐area ratio (Bergmann's Rule, Bergmann 1847), which facilitates more efficient elimination of excess body heat in warm environments (Gardner et al. 2011). Conversely, species in cold environments tend toward larger body sizes, likely in response to the need to retain heat (Bergmann 1847; Fuller et al. 2021). Relationships between temperature and species' mean body size have been detected for mammals and birds (McCain and King 2014; Hantak et al. 2021; Zimova et al. 2023), although variation in this relationship exists (Gardner et al. 2011). If thermoregulation plays a significant role in geographic trends in body size, then rapid warming over recent years could lead to shifts in body size across bird and mammal species. Such shifts could result from many processes, such as reduced foraging times under increasingly hot conditions, changes in metabolic rates, or size‐related fitness consequences of heat stress (Bowden et al. 2015; Fuller et al. 2021; McCain and King 2014).

Aridity also imposes demands on organisms that could contribute to differences in body size, as water plays a vital role in thermoregulation through evaporative cooling in endothermic vertebrates (Albright et al. 2017; Fuller et al. 2021). Although smaller body size increases cooling efficiency, it also increases the water loss rate, putting smaller body organisms at greater risk of dehydration (Albright et al. 2017; McKinley et al. 2018; Sannolo and Carretero 2019). This is further exacerbated by warmer temperatures, which can increase the need for evaporative cooling, and thereby increase an organism's water demand (McKechnie et al. 2016; McKinley et al. 2018). Consequently, dehydration risk rises with both temperature and aridity, with smaller body sizes being particularly at risk (Weathers 1981; Fuller et al. 2021). Body size patterns in endothermic vertebrates should therefore be impacted by the competing demands of changing temperature and aridity conditions (Albright et al. 2017; Fuller et al. 2021).

Human land use (HLU) intensification and expansion can amplify climate change impacts on species through simplification and homogenization of habitats, which can reduce the availability of vital microclimates that species require to avoid temperature and precipitation extremes (Williams and Newbold 2020). HLU change thus interacts with climate change by increasing species' exposure to the broader climate change trends, which can include shifts in both temperature and precipitation (Williams et al. 2022), as human‐dominated landscapes are hotter and drier compared to areas with greater natural habitat extent (Frishkoff et al. 2016). Ultimately, both climate change and HLU will alter temperatures and aridity conditions species experience, which has the potential to drive responses in body size among terrestrial vertebrates and invertebrates (Gardner et al. 2011; Schmidt and Jensen 2003; Sheridan and Bickford 2011; Van Buskirk et al. 2010).

Proximity to species' thermal and aridity tolerance limits are important factors to consider when assessing vulnerability of species to altered thermal and aridity regimes associated with climate change (Frishkoff et al. 2015; Mitchell et al. 2018; Williams and Newbold 2021). Organisms in areas already near their thermal and aridity tolerance limits could be especially susceptible to temperature and precipitation differences. Metrics like the Thermal Position Index (TPI) provide a method for generating species‐specific estimates of environmental tolerance and determine a species' proximity to its niche tolerance limits (Kerr 2020). Previous studies have found that TPI accurately predicts species range shifts in response to climate change as well as population trends due to TPI‐land‐use change interactions (Soroye et al. 2020; Williams and Newbold 2021). Among bird and mammal species experiencing conditions approaching the limits of their tolerance, changes in body size may help improve thermoregulatory performance and mitigate the effects of climate change, thereby reducing local extinction risk (Frishkoff et al. 2015; Williams and Newbold 2021).

Here, we tested for the response of body size metrics through recent shifts in temperature, aridity, and human land use extent among bird (*n* = 371) and mammal species (*n* = 281) using the Thermal (TPI) and Aridity (API) Position Indices. We assembled a global dataset of mammal and bird body mass (g) and head‐body length (mm) (Gonçalves et al. 2018; Guralnick and Constable 2010; NEON 2022; Ocampo et al. 2021; Rodrigues et al. 2019) and used it to test whether species' proximity to their thermal and aridity tolerance limits predicted changes in body size over space and time. Since smaller body size is associated with improved thermoregulatory efficiency under hot and arid conditions, we expected body size (mass, length, mass:length ratio) to decline as local conditions approached species' upper thermal (hot) and lower aridity (dry) tolerance limits. HLU is associated with hotter and drier conditions as well as increased exposure to prevailing climatic conditions, so we expected smaller body sizes to be observed in areas with greater HLU extent. In our analyses, we include two metrics of HLU, agricultural land use (ALU) and urban land use (ULU) to account for potential differences in resource availability and heat exposure (Liu et al. 2022). When conditions approach species' upper aridity (wet) tolerance limit, we expected to observe reduced effects of thermal limits on body size due to greater water availability and greater consequent opportunities for efficient thermoregulation. We did not anticipate simple temporal trends in body size at any locality, where ambient environmental conditions might more strongly determine outcomes. Nevertheless, we did anticipate generalized declines in body size because of the extent of global mean temperature increases.

## 2 Methods

### 2.1 Size Data

**FIGURE 1:**
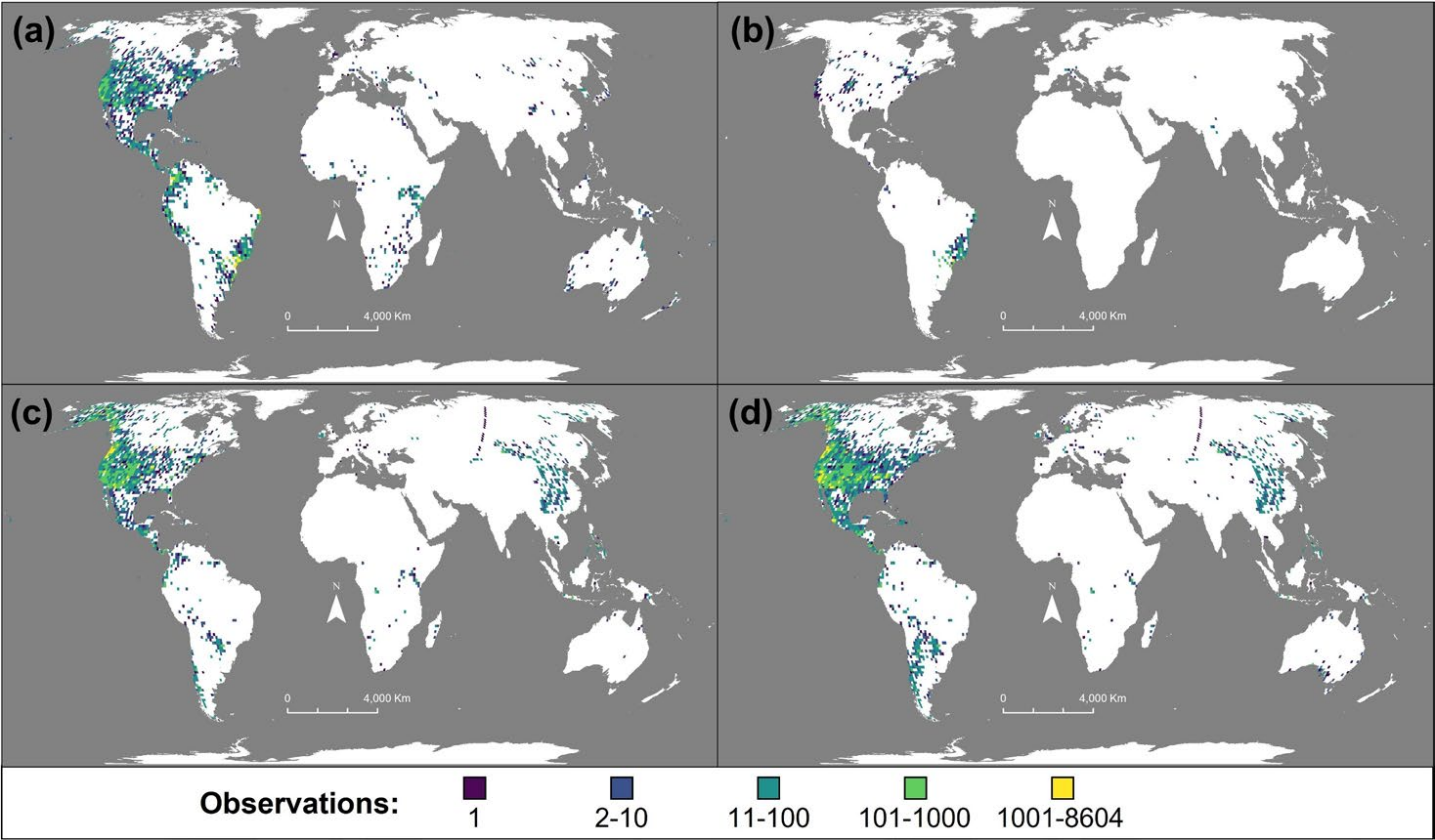
Distribution map of bird and mammal observations. (a) Bird body mass observations (*n* = 65,891, species = 254). (b) Bird body length observations (*n* = 6582, species = 32). (c) Mammal body mass observations (*n* = 162,201, species = 159). (d) Mammal body length observations (*n* = 202,261, species = 190). All maps represent the sum number of observations within ~100 km × 100 km grid cells. Map projection WGS 1984 Web Mercator (auxiliary sphere).

**FIGURE 2:**
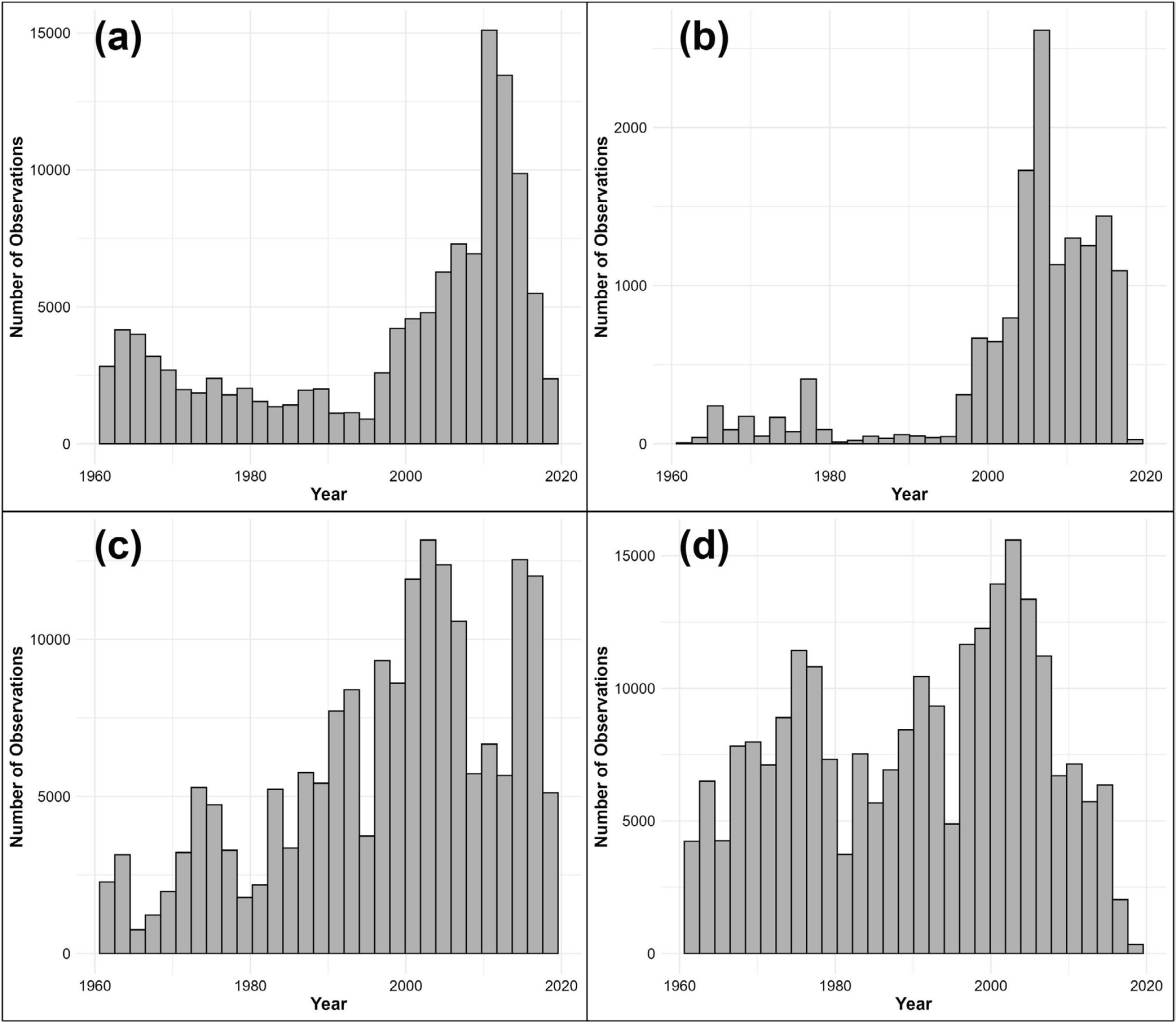
Distribution of bird and mammal observations over time. (a) Yearly number of observations of bird body mass. (b) Yearly number of observations of bird body length. (c) Yearly number of observations of mammal body mass. (d) Yearly number of observations of mammal body length.

We obtained body size data for birds and mammals (Figure 1), spanning the years 1961–2018 (Figure 2), from multiple databases: VertNet (Guralnick and Constable 2010), ATLANTIC: Data Papers (Gonçalves et al. 2018; Rodrigues et al. 2019), Ocampo et al. (2021), and the National Ecological Observatory (NEON 2022). Databases consistently used standard measurements of body size: body mass (g) and/or head‐body length (mm). We removed records that had coordinates with impossible values or coordinates where latitude and longitude are equal. We also removed records lacking date information, coordinates, body size measurements (body mass and body length), and species‐level identification. Specifically for NEON data, we only retained observations marked as non‐recaptures to avoid pseudoreplication of recaptured individuals and avoided using measurements of body length as acquiring accurate body length measurements on live specimens is difficult (Hantak et al. 2021).

We further processed the combined dataset by reassigning life stage to adult, juvenile, or unknown and sex to male, female, or unknown. Through manual inspection of the various life stage (Extended Data 10) and sex classifications (Extended Data 11), we reassigned all possible values to a determined classification when possible, and when this was not possible, a value of unknown was applied. After life stage and sex reclassifications were completed, we verified the spelling of species' scientific names using the R packages *taxize* (0.9.100) to account for erroneous entries or out of date species names (Chamberlain et al. 2020). We then passed all species names through the R package *rredlist* (0.7.1) to harmonize species names from all data sources with the species names utilized by the IUCN red list (Gearty and Chamberlain 2022). This ensured that all range maps, species TPI and API limits, and individual species observations would match in subsequent analyses.

Once all data organization and filtration processes were complete, we used the R package *terra* (1.7‐83) to extract measurements of maximum monthly temperature, aridity index, human land use proportion per quadrat, and biogeographic realm for all observations. Further, we created a grouping variable labeled Site, which was generated by grouping observations based on overlapping geographic locations (latitude and longitude).

After all necessary variables were added to our dataset, we removed juvenile observation records. For adult records, we grouped observations by species and calculated median and median absolute deviation (MAD) for measurements of body mass and body length. MAD is a robust estimate of the dispersion of sample values about the median and is resilient to the presence of outliers within a dataset. We then retained all adult and unknown age class observations that fell within five MADs of median adult mass. All observations that fell below five MADs of median adult mass or length were considered likely juvenile observations and subsequently removed. Additionally, any values that also exceeded median adult body mass or body length by five MADs or more were considered outliers that may have resulted from digitization errors and were removed.

Small sample sizes per species could yield misleading results (Hantak et al. 2021), so we removed species with fewer than 100 unique body mass measurements. This was done separately for each body size metric to generate two distinct body size datasets for each taxonomic Class. For all datasets, we added measurements of log_10_ transformed values for all body size metrics outlined above. For birds, we retained 119,183 body mass and 15,562 body length observations, and for mammals we retained 183,087 body mass and 239,600 body length observations (Table S1). Finally, we generated an estimate of species volume to surface area ratio by dividing species cubed root body mass by body length. Since lower volume to surface area ratios are associated with more efficient thermoregulation (Fuller et al. 2021) we wanted to test if climate and land use change resulted in concordant changes in both body mass and body length, resulting in changes to species volume to surface area ratios.

A comparison of the number of observations and species for birds and mammals broken down by dataset can be found in Table S8. Additionally, we evaluated our dataset for taxonomic coverage (Supporting Information) and found that the taxonomic distribution of species within our datasets is broadly representative of the diversity of orders within each class (Aves or Mammalia).

### 2.2 Environmental Data

#### 2.2.1 Thermal Position Index

To test the impacts of temperature on body size trends, we used the Thermal Position Index (TPI), a scaled metric that identifies a species' relative position within its realized thermal niche space (Kerr 2020). TPI is calculated for a species at a given temperature using the formula:

$$P=\frac{1}{t}\;\sum_{i=1}^{t}\frac{\left(N_{m}-N_{\min}\right)}{\left(N_{\max}-N_{\min}\right)}$$

where *P* is the measured TPI value assessed over a time period of *t* units, *N*_m_ is the temperature experienced by the species for each time unit, *N*_min_ and *N*_max_ are the absolute minimum and absolute maximum temperatures experienced across a species range for the matching time unit *t*. TPI values of 0 and 1 represent temperatures that are equal to a species lower and upper thermal tolerance limits, respectively. TPI limits are not intended to serve as critical thermal limits, but rather can be used to estimate species environmental tolerances and measure change in environmental conditions relative to derived limits. Therefore, TPI values that exceed 1 or fall below 0 represent conditions that exceed species theoretical thermal tolerance limits.

To generate estimates of species upper (*N*_max_) and lower (*N*_min_) thermal niche limits, we collected historical monthly maximum and minimum temperature datasets from the WorldClim database (Fick and Hijmans 2017). We then used species range maps (IUCN 2017; BirdLife International and Handbook of the Birds of the World 2021) to extract the minimum and maximum temperature values across each species range distribution for the Years 1961–1975. This range of dates serves as a baseline period prior to the onset of rapid climate change (Soroye et al. 2020). We then calculated the mean maximum and minimum temperature for each month for all years between 1961 and 1975 and generated monthly lower and upper thermal niche limits for each species. A more detailed description of the methodologies used in generating upper and lower thermal niche limits can be found in the Supporting Information (Supplementary Text).

To generate TPI values for our observations, we extracted the monthly maximum temperature for the date and location of each individual observation (*N*_s_). For each observation, we matched the species and month of observation to select the upper and lower thermal limits (*N*_min_ and *N*_max_) to be used in the TPI equation. Using these limits, we then calculated TPI for each observation within the datasets. A detailed process of how thermal tolerance limits were generated for each species is provided in the Supporting Information.

#### 2.2.2 Aridity Position Index (AI)

The Aridity Index (AI) provides a measurement of environmental moisture availability that considers both precipitation (PPT) and evapotranspiration (PET) (Middleton and Thomas 1997), with increasing AI values representing higher levels of environmental moisture. We collected monthly precipitation and potential evapotranspiration data from TerraClimate (Abatzoglou et al. 2018) at a gridded resolution of ~21 km and calculated the monthly aridity values in R. Finally, we averaged monthly aridity values to generate maps of yearly average aridity for 1961–2018. Areas covered in snow did not contain values for potential evapotranspiration, which resulted in variations in regional coverage of aridity values for each month.

We followed the same procedure to generate API limits as was used for calculating TPI limits (Supporting Information), with an adjustment to account for extreme AI values. Due to extremely low PET values, it is possible to generate AI values that are disproportionate to regional AI values. To account for this when extracting AI values for each species, we identified outlier AI values and calculated replacement values. We considered AI values outliers if they were greater than the third quartile of range‐wide AI values plus 1.5 times range‐wide AI interquartile range, or lower than the first quartile of range‐wide AI values minus 1.5 times range‐wide AI interquartile range. When outlier values were identified, we replaced them with the upper or lower calculated cutoff values used to determine outlier datapoints. In total, 2.2% of upper AI limits were adjusted through this method for 67 out of 652 species.

After accounting for extreme AI values, all downstream calculations were conducted in the same manner as generating TPI limits. We first extracted AI values for the month and year of each observation in the dataset. Then, using the TPI equation, we calculated API values for all observations using the matching AI tolerance limits for the corresponding species and month of observation.

#### 2.2.3 Land‐Use

We calculated percent human land use per quadrat (5 km by 5 km) from 1961 to 2018 to evaluate how anthropogenic land use modification affected how species respond to climate change. Using R Studio and the *terra* (1.7‐83) package, we used a historic land use dataset accessed from PANGEA to create two estimates of human land use (Winkler et al. 2020). We first summed percent land use for cropland and pastureland to generate an estimate of percent agricultural land use (ALU) at a gridded resolution of 5 km by 5 km at a global scale for the years 1961–2018. Then we generated an estimate of urban land use (ULU) following the same procedure. Values closer to zero indicate predominantly unmodified land use, while values closer to one represent an increasing prevalence of human‐related land uses. In addition to human land use, we included biogeographic realm in our datasets to control for potential differences across regions. All calculations and data generation outlined for TPI, API, ALU, and ULU were carried out in R (R Core Team 2022; 4.3.1), and all scripts can be accessed through the Supporting Information: Data and Materials section.

### 2.3 Statistical Analyses

All statistical analyses were conducted using R (R Core Team 2022). We investigated how measures of body mass, body length, and mass–length ratio among bird and mammal species changed spatially and interannually relative to spatial and temporal gradients of climate and land use. Phylogenetic distance may influence adaptive responses in species, with species that are phylogenetically closer being more likely to exhibit a similar response due to more recent shared evolutionary history (Davis et al. 2010). To control for the potential effect of phylogenetic clustering of responses, we constructed phylogenetic informed generalized linear mixed models (PGLMM) using the *phyr* (1.1.0) package in R (Li et al. 2020). We fitted separate PGLMM models for birds and mammals to determine the specific responses of each Class to climate change. For each taxonomic class, we fitted models with a Gaussian distribution under a Bayesian framework. Model variables included TPI, API, ALU, and ULU, as level one fixed effects, and interaction effects of TPI with API, TPI with ALU, and TPI with ULU. Additionally, we included year as a covariate to account for potential temporal trends in body size metrics. All model variables were *z*‐score transformed to aid interpretation and comparison of model coefficients and improve model sampling efficiency.

We included four random intercept terms and two uncorrelated random slope terms in our model. Random intercept terms were fit for Biogeographic Realm, site of observation, species binomial, and phylogenetic distance. Season was originally included as an additional random intercept term but was found to not account for significant variance in our models and was removed. For each model, we determined the relationship of the dependent body size variable against each level one fixed effect (TPI, API, ALU, ULU, Year) through the combination of two slopes determined by the random effects of species identity (Binomial). The first random slope was determined by species identity assuming that species are independent. To account for the nonindependence of phylogeny, we generated covariance matrices for all species in each dataset using phylogenies for mammals (“DNA‐only”) and birds (Hackett backbone) (Jetz et al. 2012; Upham et al. 2019). The second slope was then determined using species identity while accounting for phylogenetic nonindependence through the inclusion of phylogenetic covariance matrices. Default integrated nested Laplace approximation (INLA) priors were used.

All models were inspected to validate that they met the assumptions for PGLMM. We tested all models for the presence of outliers which may be overly influential on model estimates. For each model, less than 1% of observations were detected as potential outliers which may be exerting high leverage on our results. We removed the observations from the datasets and reran each model. We found that all model estimates were robust to the removal of these data. Tests of multicollinearity of predictor variables found no evidence of problematic collinearity and are reported in Tables S2–S7 (variance inflation factor: VIF). To account for potential spatial clustering influencing our results, we inspected all models for signs of spatial autocorrelation. Using spline correlograms, we tested each model for correlation of model residuals based on geographic distance between observation locales (Latitude and Longitude). We detected no significant signals of spatial autocorrelation (Figure S1a–d). We further inspected our models for signs of temporal autocorrelation effects. We used the Durbin–Watson test on 1000 random samples of model residuals across all years for each model. We found that the models showed no evidence for temporal autocorrelation between 84.6% and 95.5% of the time, and model residuals plotted over time show no overall trend (Figure S2a–d). We also tested each species for signs of temporal autocorrelation. For each species in each model, we ran 100 iterations of the above residual extraction and analysis protocol. We then averaged each species' Durbin–Watson statistics and significance values to test if any species' body size trend exhibited significant temporal autocorrelation. Species that were identified as showing temporal autocorrelation were removed from the datasets and the models were rerun. Comparison of models with the removed species showed no changes to model estimates or significance. We therefore report results from models with all species included.

## 3 Results

In total, we generated datasets containing 119,183 bird body mass observations (370 species), 15,562 bird body length observations (63 species), 183,087 mammal body mass observations (203 species), and 239,600 mammal body length observations (276 species). Records for both bird and mammal body size measurements spanned 58 years (1961–2018; Figure 2) and all major biogeographic realms except for the Antarctic (Figure 1). Although not all bird and mammal taxonomic orders are represented within our dataset, the taxonomic orders that are present were found to be proportionally represented by the taxonomic coverage of species within our dataset (Supporting Information: Supplementary Text; Figure S4).

### 3.1 Environmental Relationships With Body Size

**FIGURE 3:**
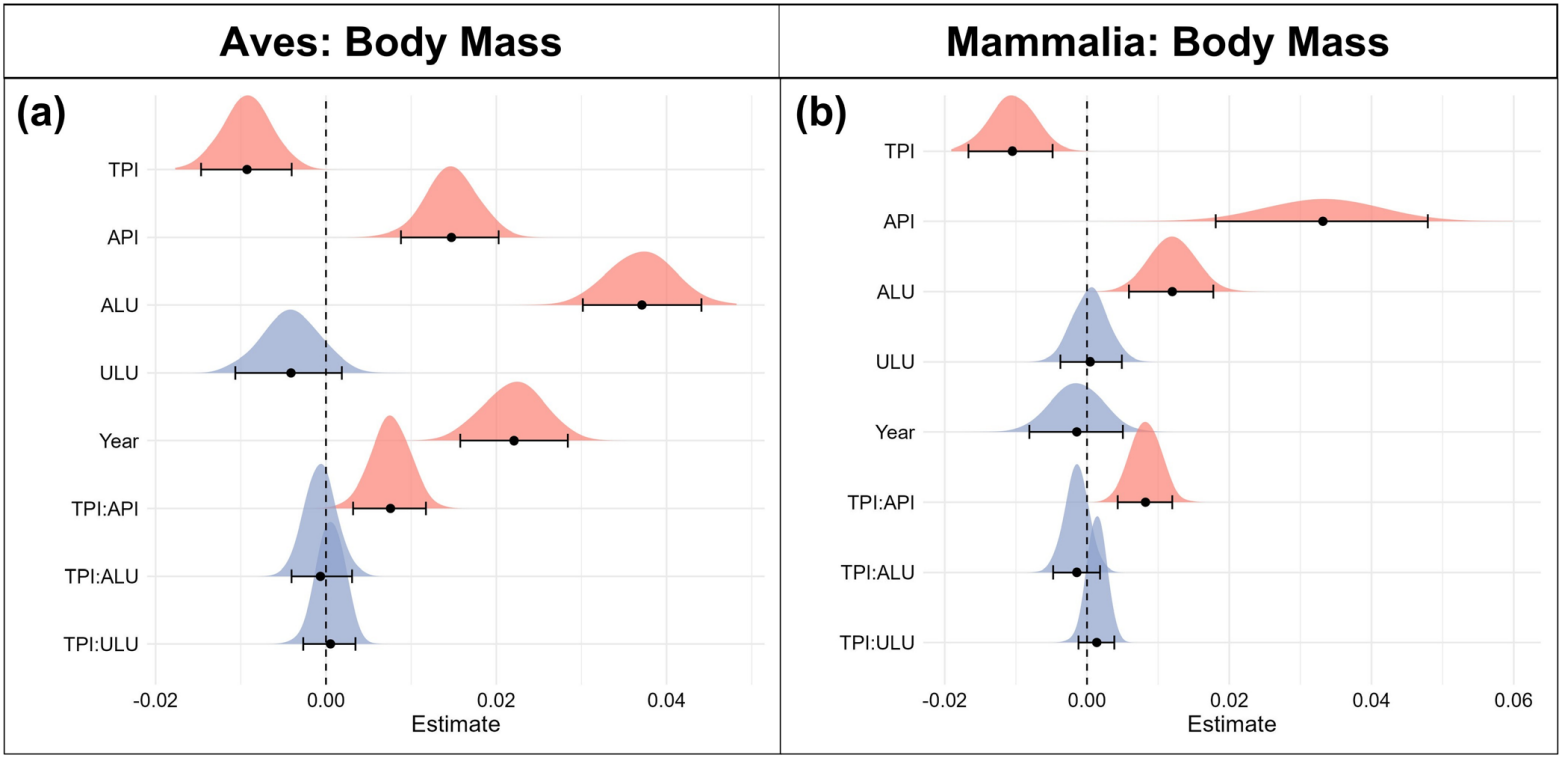
Response of log_10_ body mass to environmental variables. (a) Coefficient estimates of model variables effect on Bird (Aves) log_10_ body mass (partial *R* ^2^ = 0.929) (Table S2). (b) Coefficient estimates of model variables effect on Mammal (Mammalia) log_10_ body mass (partial *R* ^2^ = 0.949) (Table S5). Coefficient estimates represent the effect that a 1 standard deviation increase has on *z*‐scaled log_10_ body mass. Error bars represent 95% credible intervals of model estimates. Density plots display the complete distribution of model estimates. Red density plots indicate significant variables based on 95% credible intervals not overlapping 0, while blue density plots are used to indicate nonsignificant variables.

**FIGURE 4:**
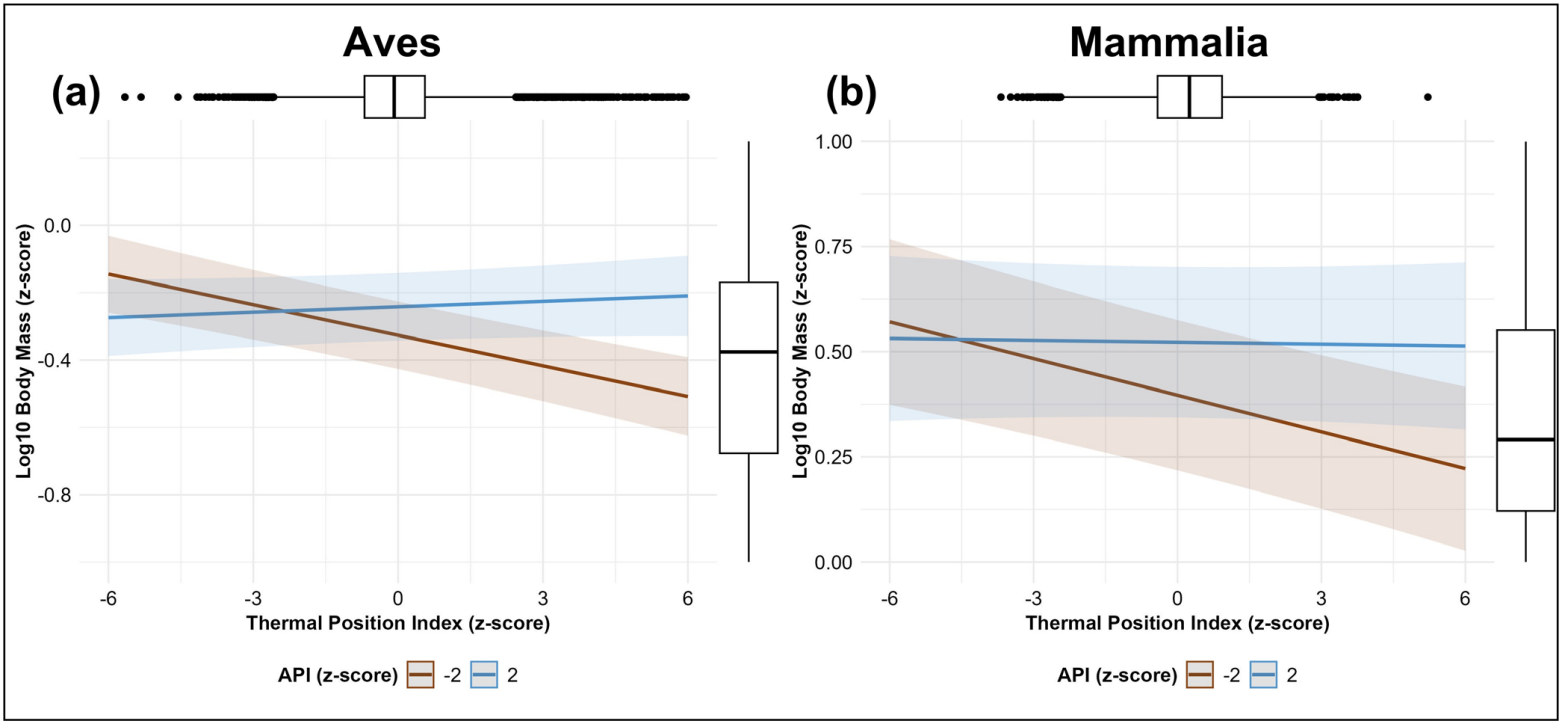
Response of log_10_ body mass to interaction of TPI and API (a) Interaction effect of TPI and API on log_10_ body mass of bird species. All variables are presented as *z*‐transformed values. Here we show estimated body mass relationships to TPI where API is equal to −2 standard deviations (brown; dryer conditions) or +2 standard deviations (blue; wetter conditions) of the values across all populations. Shaded areas display 95% credible intervals. Boxplots show the distribution of *z*‐score‐standardized log_10_ body mass values of terrestrial vertebrates and the *z*‐score‐standardized TPI. (b) Interaction effect of TPI and API on log_10_ body mass of mammal species. All variables are presented as *z*‐transformed values. Here we show estimated body mass relationships to TPI where API is equal to −2 standard deviations (brown; dryer conditions) or +2 standard deviations (blue; wetter conditions) of the values across all populations. Shaded areas display 95% credible intervals. Boxplots show the distribution of *z*‐score‐standardized log10 body mass values of terrestrial vertebrates and the *z*‐score‐standardized TPI.

TPI, API, and ALU predicted global geographical and interannual variation in body mass for bird (Table S2) and mammal species (Table S5). Smaller body masses were associated with elevated TPI values for both birds (*β* = −0.0094, 95% CI = −0.0146 to −0.0042; Figure 3a) and mammals (*β* = −0.0106, 95% CI = −0.0165 to −0.0048; Figure 3b). Both birds and mammals had larger body masses in areas with higher API (wetter) values (birds: *β* = 0.0147, 95% CI = 0.0089–0.0205; mammals: *β* = 0.0329, 95% CI = 0.0180–0.0479) and greater ALU extent (birds: *β* = 0.0370, 95% CI = 0.0303–0.0437; mammals: *β* = 0.0012, 95% CI = 0.0056–0.0180) (Figure 3a,b). We observed no significant effect of ULU on bird or mammal body mass. Our results also show a significant positive interaction between TPI and API for both birds (*β* = 0.0076, 95% CI = 0.0033–0.0120) (Figure 3a) and mammals (*β* = 0.0083, 95% CI = 0.0042–0.0124) (Figure 3b), indicating that the negative relationship between body mass measurements and TPI gradients is greater when species are closer to their lower API (dry) limits (Figure 4a,b). No interaction between either ALU or ULU and TPI was detected for either taxonomic group in terms of effects on body mass.

**FIGURE 5:**
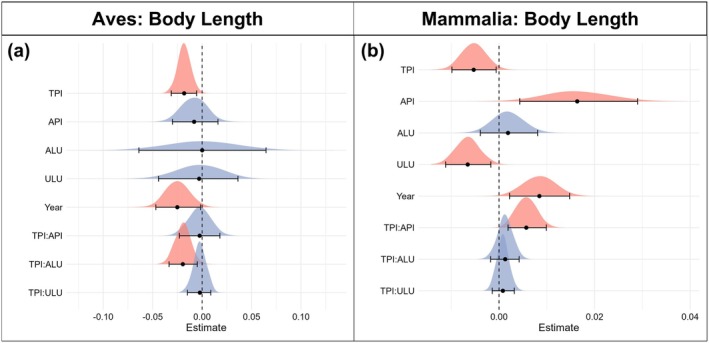
Response of log_10_ head‐body length to environmental variables. (a) Coefficient estimates of model variables effect on Bird (Aves) log_10_ head‐body length (partial *R* ^2^ = 0.941) (Table S3). (b) Coefficient estimates of model variables effect on Mammal (Mammalia) log_10_ head‐body length response to changes in model variables (partial *R* ^2^ = 0.936) (Table S6). Coefficient estimates represent the effect that a 1 standard deviation increase has on *z*‐scaled log_10_ body length. Error bars represent 95% credible intervals of model estimates. Density plots display the complete distribution of model estimates. Red density plots indicate significant variables based on 95% credible intervals not overlapping 0, while blue density plots are used to indicate nonsignificant variables.

Patterns of body length response to TPI were consistent between birds and mammals; however, body length responses to API, ALU, and ULU were more variable and differed between birds (Tables S3) and mammals (Tables S6). Shorter body lengths for both bird and mammal species were associated with higher TPI values (birds: *β* = −0.0181, 95% CI = −0.0302 to −0.0062; mammals: *β* = −0.0052, 95% CI = −0.099 to −0.0005) (Figure 5a,b). Bird body length was not significantly related to API conditions, while mammals showed a positive relationship (*β* = 0.0168, 95% CI = 0.0039–0.0298) (Figure 5b). Body length measurements in birds were not related to ALU or ULU; however, shorter body lengths in mammals were associated with increased ULU (*β* = −0.0067, 95% CI = −0.0114 to −0.0019) (Figure 5b). TPI was found to negatively interact with ALU for birds (*β* = 0.0020, 95% CI = −0.0344 to −0.0053), indicating that greater extents of agricultural land use exacerbate the impacts of TPI on body length. For mammals, we observed a positive interaction between TPI and API (*β* = 0.0058, 95% CI = 0.0017–0.0099) (Figure 5b) which means that the relationship between body length and TPI is less pronounced for species occupying environments closer to their upper API limits (wetter).

**FIGURE 6:**
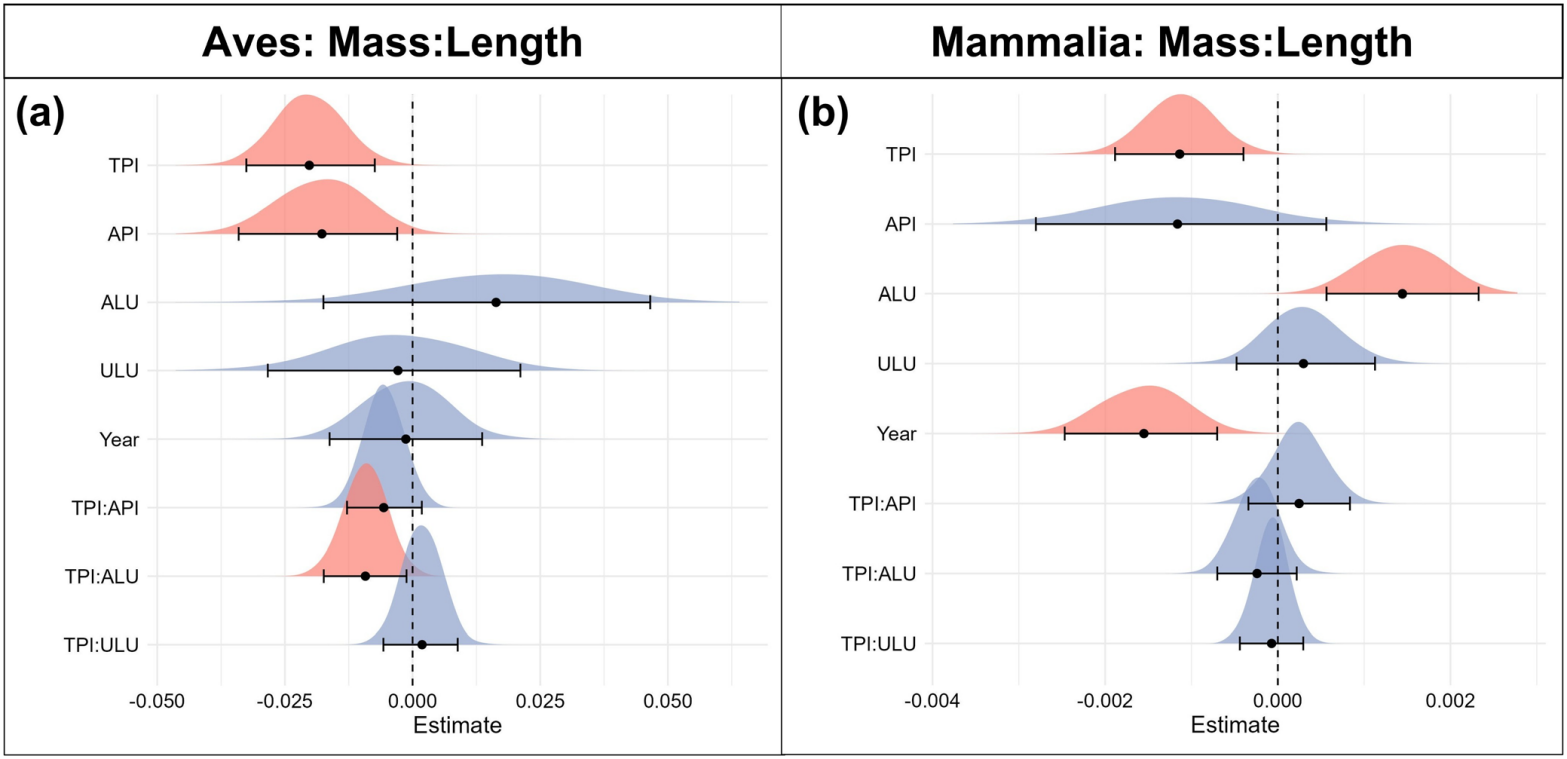
Response of body mass to length ratio to environmental variables. (a) Coefficient estimates of model variables effect on Bird (Aves) ∛Mass/Length (partial *R* ^2^ = 0.977) (Table S4). (b) Coefficient estimates of model variables effect on Mammal (Mammalia) ∛Mass/Length (partial *R* ^2^ = 0.868) (Table S7). Coefficient estimates represent the effect that a 1 standard deviation increase has on *z*‐scaled body mass to length ratio. Error bars represent 95% credible intervals of model estimates. Density plots display the complete distribution of model estimates. Red density plots indicate significant variables based on 95% credible intervals not overlapping 0, while blue density plots are used to indicate nonsignificant variables.

Analyses of body mass to length ratio were conducted as an estimate for species volume to surface area ratio, which is known to impact thermoregulatory efficiency. We found that body mass to length ratios varied in response to climate and land use. Lower body mass to length ratios were observed for both birds (*β* = −0.0202, 95% CI = −0.03305 to −0.0073) (Figure 6a) and mammals (*β* = −0.0012, 95% CI = −0.0019 to −0.0004) (Figure 6b) when conditions were closer in proximity to species' upper TPI limits. Similar patterns were found for API in birds, with lower body mass to length ratios associated with higher API (wetter) conditions (*β* = −0.0179, 95% CI = −0.03537 to 0.0022) (Figure 6a). API did not predict body mass to length ratios among mammal species. However, body mass to length ratios for mammals did display a positive response to ALU gradients (*β* = 0.0015, 95% CI = 0.0006–0.0023) (Figure 6b), a trend that was absent among birds. The only significant interaction observed was a negative relationship between TPI and ALU for birds (*β* = −0.0092, 95% CI = −0.0168 to −0.0015). This indicates that, for birds, the relationship between body mass: length ratios and TPI is more pronounced in regions with a greater extent of agricultural land use.

Effects of TPI, API, and HLU on body size for both mammal and bird species, respectively, were independent of time‐related trends (see Supporting Information for detail). Model results excluding the effect of year can be found in Tables S9 and S10.

## 4 Discussion

At a global extent, birds and mammals exhibited smaller body sizes in environments closer to species' upper thermal limits (Figures 3, 4a, and 5). This pattern is consistent with the hypothesis that body size reflects differences in thermoregulatory efficiency (Fuller et al. 2021; Weathers 1981), with smaller‐bodied individuals exhibiting a greater capacity to tolerate hotter environmental conditions. Limitations on heat tolerance are known to impose greater risks of chronic sub‐lethal effects of heat stress on large‐bodied birds and mammals (Conradie et al. 2019; McCain and King 2014), which can increase the severity of mortality events in response to extreme temperatures (McKechnie and Wolf 2010; Welbergen et al. 2007). This suggests birds and mammals are responding plastically or adapting to rising temperatures through reductions in body sizes over generations to improve thermoregulation in extreme conditions.

The association of smaller body size with environments closer to species' upper thermal tolerance limits may also be driven by indirect pressures associated with climate change. Reductions in food quality or availability may help explain observed associations of smaller body size in endothermic vertebrates within hotter environments (Brivio et al. 2016; Oswald et al. 2021). These indirect effects of warming trends may consequently constrain individual growth rates in addition to the direct effects related to requirements of thermoregulation, reinforcing the relationship between smaller body sizes and hotter environments in birds and mammals (Andreasson et al. 2018; Speakman and Król 2010). However, long term declines in body size can arise in populations of endothermic vertebrates independent of these resource quality or quantity processes (Mason et al. 2014). Hotter temperatures push species towards their upper thermal tolerance limits and are associated with reductions in foraging duration and effectiveness due to increasing thermoregulatory limitations (Mason et al. 2014; Oswald et al. 2021). Individuals must spend more time engaging in thermoregulatory behaviors which can reduced food provisioning. During critical growth periods reductions in resource provisioning could result in smaller individuals within environments nearer the boundaries of species' upper thermal tolerances (Dubos et al. 2019; Hantak et al. 2021). This process is supported by the consistent relationships we have uncovered between smaller body sizes and hotter environments in bird and mammal species.

Smaller body sizes were observed for birds and mammals the closer species were to their aridity tolerance limits (Figures 3, 4b, and 5a), with larger body sizes observed toward the wetter boundaries of species' realized niches. This association is consistent for body mass in birds and both body mass and body length in mammals. API did not predict body mass to body length ratio for mammals, which indicated that body mass and body length covaried proportionally across aridity gradients. Water availability and body size both play vital roles in thermoregulation for endothermic vertebrates (McKechnie et al. 2016; Sannolo and Carretero 2019), particularly when water is limited, and species must rely on dry heat exchange (Weathers 1981; Mitchell et al. 2018). Individuals with larger body sizes benefit from increased water availability by being able to store proportionally more water (Fuller et al. 2021), improving tolerance of hotter temperatures through sustained evaporative cooling with lower risk of dehydration (Mitchell et al. 2018). This would result in larger body sizes across birds and mammals being associated with species' upper API (wet) limits.

There is proportionally greater water availability in environments where API approaches its highest values for species (wet limit), and primary or secondary productivity will tend to increase in such areas (Golodets et al. 2015). Since body size can be limited by environmental productivity and resource availability (Yom‐Tov and Geffen 2006), increased productivity may increase body sizes in environments closer to species' upper API (wet) limits. However, body size has been found to decline with increasing temperature in birds and mammals due to limitations in thermoregulatory capacity rather than changes in resource availability or quality (Mason et al. 2014; Oswald et al. 2021), and we would expect differences in water availability to impact the response of body size to temperature.

The smallest body sizes were observed in areas where species occurred near their upper thermal (hot) and lower aridity (dry) limits. Changes in water availability across species' aridity tolerance range is associated with body sizes, at least in part, through physiological demands of thermoregulation. This response is consistent with requirements for increased demand for thermoregulatory efficiency since dehydration risks in progressively hotter environments increase as species approach their dry tolerance limits. Previous studies have found that constraints on body size in response to changes in temperature for bird species depend on the position of populations within their thermal tolerance range (Dubos et al. 2019). Here, we show that for birds and mammals, relationships of body size to thermal niche position varies based on species' relative aridity niche position.

We found positive associations between body size and the extent of agricultural land use for both birds and mammals. Body mass in both birds and mammals was found to be heavier at higher extents of agricultural land use, counter to our predictions. Measurements of species' body sizes in heavily modified landscapes may have been biased towards species that successfully tolerate the presence of extensive habitat modification. Such species are more likely to benefit from increased resource availability afforded by human land uses (Li et al. 2022), as well as from increased productivity with wetter environments, potentially driving increases in body sizes in areas near species' upper API (wetter) limits (Figures 3, 4b, and 5a). Urban land use extent was found to negatively impact body length in mammals, although no interaction between ALU or ULU and TPI was observed. Smaller body size in mammals within urban environments has been observed (Guralnick et al. 2020; Ofori et al. 2025) and is attributed to increased thermoregulatory demands of urban areas that are often hotter than surrounding rural and natural environments.

Contrary to mammals, we observed a significant negative interaction between ALU and TPI in birds for body length measurements and mass:length ratios. This indicates that birds are shorter and have smaller overall mass:length ratios when they are closer in proximity to their upper thermal tolerance limits and occupy regions with a greater extent of agricultural land use. The increased association of smaller body sizes with elevated TPI in regions with greater ALU extent is likely driven by agricultural land uses amplifying exposure to extreme temperatures by limiting availability of cooler microclimates (Hardwick et al. 2015; Williams and Newbold 2021). While land use and warming temperatures can interact to cause populations to decline more rapidly (Williams et al. 2022; Williams and Newbold 2021), our findings suggest that, for birds, changes in body size can also respond to this interaction.

The frequency and severity of extreme heat events is increasing with human‐caused climate change (Perkins‐Kirkpatrick and Lewis 2020), altering aridity patterns (IPCC 2022), and intensifying selective pressures across taxa (Boyles et al. 2011; Dubos et al. 2019; Fuller et al. 2021). A study looking at the response of mammals to climate change found that larger species were more likely to respond through range shifts, declines in abundance, and regional extirpations compared to smaller species (McCain and King 2014). Further, during the Pleistocene, changes in climate have been linked to the extinction of megafauna species, while smaller‐bodied mammal species showed no change in extinction rates (Barnosky et al. 2004; Blois et al. 2010). Changes in body sizes of endothermic vertebrates over time are therefore likely to affect species persistence of populations as anthropogenic‐driven climate change and land uses continue to shift (Sheridan and Bickford 2011). For both bird and mammal species, we found consistent spatial and interannual associations of smaller body size metrics with increasingly hot and dry conditions relative to species' tolerance limits. Relationships reported here suggest that body size is likely to decline for birds and mammals experiencing rising temperatures and increasingly dry conditions with continued climate change. However, these species may have limited physiological capacity to respond to differences in temperature, aridity, and human land uses, leading to variability in body size trends, despite their relative consistency to date.

Global differences in body size among bird and mammal species show broadly convergent relationships in response to spatial and interannual differences in temperature and aridity. Other responses to rapid global changes include geographical range shifts and differences in phenological timing (Hodgson et al. 2012; Rudolf and Singh 2013), which track species' niche requirements but that may not necessarily relate to their functional traits (MacLean and Beissinger 2017; Sirois‐Delisle et al. 2024). Body size, however, is strongly linked to many fundamental life history traits and processes, including reproductive rates, growth rate, foraging capacity, and even trophic interactions (Berger et al. 2008; De Roos et al. 2003; Peters 1983). Changes in body size arising from climate changes could change species' resource demands, rates of population growth, and result in altered population and community dynamics (De Roos et al. 2003). Changes in body size also have the potential to affect body size variability within species, as body size variation is more constrained at smaller body sizes (Hallgrimsson and Maiorana 2008). In contrast, increased climate variability has been associated with greater body size variation within species (Read et al. 2018). This highlights the potential for conflicting pressures that climate change may impose on body size adaptation among terrestrial vertebrates. Furthermore, because species' thermal and aridity tolerance limits are unique, shifts in body sizes will likely vary among species that experience similar rates of warming or drying. Predicting the consequences of such changes will be challenging among groups for which long‐term observations for body size responses have not yet been assembled, limiting capacities to predict potential changes to community dynamics and ecosystem functioning among interacting species (Woodward et al. 2005).

Predicting how species respond to climate change is vital for developing conservation and management strategies that anticipate changes in community composition (Boukal et al. 2019; Rudolf and Singh 2013). Both range and phenological shifts in response to changing climatic conditions are widespread across taxa (Boyles et al. 2011; McCain and King 2014; Riddell et al. 2021), but understanding why body sizes change in response to rising temperatures is far less well characterized (Hantak et al. 2021; Van Buskirk et al. 2010; Youngflesh et al. 2022). Here, we show that bird and mammal body size varies with species' climatic niche position. In regions that are hotter and dryer relative to species' realized niche limits, body sizes are substantially smaller. Aridity and agricultural land use extent were both associated with larger body size measurements among birds and mammals, independent of warming trends. Such effects can reflect both the direct consequences of environmental changes on species' capacities for thermoregulation and indirect effects on local resource availability. Body size affects many other traits, from physiological to ecological. Yet, this fundamental species trait appears to be sensitive to environmental conditions in similar ways among a variety of bird and mammal species observed across biogeographical realms. The physiological and ecological consequences of such a relationship may prove as pervasive as the effects of geographical range and phenological shifts that are now relatively well recognized.

## Author Contributions

**Matthew J. Watson:** conceptualization, data curation, formal analysis, investigation, methodology, validation, visualization, writing – original draft, writing – review and editing. **Jeremy T. Kerr:** conceptualization, funding acquisition, project administration, resources, supervision, writing – original draft, writing – review and editing.

## Conflicts of Interest

The authors declare no conflicts of interest.

## Supporting information


**Figure S1.** Spline correlograms testing for spatial autocorrelation of model residuals. No evidence of spatial autocorrelation is observed for (a) bird body mass model (Table S2), (b) mammal body mass model (Table S5), (c) bird body length model (Table S3), (d) mammal body length model (Table S6), (e) bird mass:length model (Table S4), (f) mammal mass:length model (Table S7). Distance reported in kilometers.
**Figure S2.** Plot of model residuals against year for (a) Bird body mass, 846 out of 1000 samples showed no temporal autocorrelation (84.6%). (b) Mammal body mass, 951 out of 1000 samples showed no temporal autocorrelation (95.1%). (c) Bird body length, 955 out of 1000 samples showed no temporal autocorrelation (95.5%). (d) Mammal body length, 947 out of 1000 samples showed no temporal autocorrelation (94.7%). (e) Bird mass:length ratio, 865 out of 1000 samples showed no temporal autocorrelation (86.5%). (f) Mammal mass:length ratio, 949 out of 1000 samples showed no temporal autocorrelation (94.9%). Trend line fit using GAM (Residual ~ Year) to allow for potential nonlinear patterns (includes confidence intervals).
**Figure S3.** Plot of model posterior predictive check for (a) Bird body mass (Table S2), (b) Mammal body mass (Table S5), (c) Bird body length (Table S3), (d) Mammal body length (Table S6), (e) Bird mass:length ratio (Table S4), (f) Mammal mass:length ratio (Table S7). 1000 simulated *y* values were generated from each model (y_sim) and compared to 1000 random sampled model response variable (*y* values).
**Figure S4.** Taxonomic coverage results. Y axis represents the log_10_ transformed percentage each taxonomic order represents within the analyzed dataset. X axis represents the log_10_ transformed percentage of species each taxonomic order represents for the taxonomic classes Aves and Mammalia. The percentage of species distribution across the orders in our dataset are strongly correlated with the proportional representation of each Order within the taxonomic Classes of Aves and Mammalia (Pearson’s *R* = 0.705, 95% CI = 0.437–0.858, *p* = < 0.001).
**Table S1.** Displays the number of observations, species, geographic sites, and biogeographic realms for each body size metric for both birds (Aves) and mammals (Mammalia).
**Table S2.** Reporting the Phylogenetic Generalized Linear Mixed Model results for bird log_10_ body mass. The model was fit under a Bayesian framework (partial *R* ^2^ = 0.930). Residual variance = 0.0774 (SD = 0.278). Beta estimates reported with 95% Credible Intervals and probability of direction (pd). Random effect variance reported with standard deviation and 95% Credible Intervals. A variable followed by |Binomial indicates a random slope effect by species, while |Binomial *P* indicates random slope effects with a control for phylogenetic correlation. Variance inflation factor (VIF) reported for each variable, showing no issues of variable collinearity.
**Table S3.** Reporting the Phylogenetic Generalized Linear Mixed Model results for bird log_10_ body length. The model was fit under a Bayesian framework (partial *R* ^2^ = 0.941). Residual variance = 0.0658 (SD = 0.2564). Beta estimates reported with 95% Credible Intervals and probability of direction (pd). Random effect variance reported with standard deviation and 95% Credible Intervals. A variable followed by |Binomial indicates a random slope effect by species, while |Binomial *P* indicates random slope effects with a control for phylogenetic correlation. Variance inflation factor (VIF) reported for each variable, showing no issues of variable collinearity.
**Table S4.** Reporting the Phylogenetic Generalized Linear Mixed Model results for bird log_10_ body mass:length ratio (∛Mass/Length). The model was fit under a Bayesian framework (partial *R* ^2^ = 0.977). Residual variance = 0.0278 (SD = 0.1678). Beta estimates reported with 95% Credible Intervals and probability of direction (pd). Random effect variance reported with standard deviation and 95% Credible Intervals. A variable followed by |Binomial indicates a random slope effect by species, while |Binomial *P* indicates random slope effects with a control for phylogenetic correlation. Variance inflation factor (VIF) reported for each variable, showing no issues of variable collinearity.
**Table S5.** Reporting the Phylogenetic Generalized Linear Mixed Model results for mammal log_10_ body mass. The model was fit under a Bayesian framework (partial *R* ^2^ = 0.949). Residual variance = 0.0555 (SD = 0.2355). Beta estimates reported with 95% Credible Intervals and probability of direction (pd). Random effect variance reported with standard deviation and 95% Credible Intervals. A variable followed by |Binomial indicates a random slope effect by species, while |Binomial *P* indicates random slope effects with a control for phylogenetic correlation. Variance inflation factor (VIF) reported for each variable, showing no issues of variable collinearity.
**Table S6.** Reporting the Phylogenetic Generalized Linear Mixed Model results for mammal log_10_ body length. The model was fit under a Bayesian framework (partial *R* ^2^ = 0.936). Residual variance = 0.0700 (SD = 0.2646). Beta estimates reported with 95% Credible Intervals and probability of direction (pd). Random effect variance reported with standard deviation and 95% Credible Intervals. A variable followed by |Binomial indicates a random slope effect by species, while |Binomial *P* indicates random slope effects with a control for phylogenetic correlation. Variance inflation factor (VIF) reported for each variable, showing no issues of variable collinearity.
**Table S7.** Reporting the Phylogenetic Generalized Linear Mixed Model results for mammal log_10_ body mass:length ratio (∛Mass/Length). The model was fit under a Bayesian framework (partial *R* ^2^ 0.868). Residual variance = 0.0010 (SD = 0.0313). Beta estimates reported with 95% Credible Intervals and probability of direction (pd). Random effect variance reported with standard deviation and 95% Credible Intervals. A variable followed by |Binomial indicates a random slope effect by species, while |Binomial *P* indicates random slope effects with a control for phylogenetic correlation. Variance inflation factor (VIF) reported for each variable, showing no issues of variable collinearity.
**Table S8.** Comparison of number of observations from original datasets to filtered datasets. Filtered datasets contain all observations before filtering species based on the requirement of 100 observations for inclusion.
Table S9. Reporting the Phylogenetic Generalized Linear Mixed Model results for bird log_10_ body mass, length, and mass:length ratio (∛Mass/Length) with the year variable term removed. These models highlight that our models (Tables S2–S4) are robust to the inclusion of year as a covariate term. The models were fit under a Bayesian with Beta estimates reported with 95% Credible Intervals. All variables and random effects are conserved between the main models (Tables S2–S4) and the models reported here. A variable followed by |Binomial indicates a random slope effect by species, while |Binomial *P* indicates random slope effects with a control for phylogenetic correlation.
**Table S10.** Reporting the Phylogenetic Generalized Linear Mixed Model results for mammal log_10_ body mass, length, and mass:length ratio (∛Mass/Length) with the year variable term removed. These models highlight that our models (Tables S5–S7) are robust to the inclusion of year as a covariate term. The models were fit under a Bayesian with Beta estimates reported with 95% Credible Intervals. All variables and random effects are conserved between the main models (Tables S5–S7) and the models reported here. A variable followed by |Binomial indicates a random slope effect by species, while |Binomial *P* indicates random slope effects with a control for phylogenetic correlation.

## Data Availability

The datasets and R scripts that support the findings of this study are openly available in Figshare at https://doi.org/10.6084/m9.figshare.28418666 and GitHub at https://github.com/MacroEcoMatt/Body_Size_Climate_Change. Climate and land use datasets were obtained from WorldClim at https://worldclim.org/data/monthlywth.html (temperature) and https://doi.org/10.7923/G43J3B0R (precipitation and evapotranspiration data). Human land use data were obtained from PANGAEA at https://doi.org/10.1594/PANGAEA.921846. Species size data were obtained from Vertnet at https://doi.org/10.7946/P2K01C and https://doi.org/10.7946/P2TG68, NEON (National Ecological Observatory Network) at https://data.neonscience.org/dataproducts/DP1.10072.001/RELEASE2022, Ecology at https://doi.org/10.1002/ecy.2647 and https://doi.org/10.1002/ecy.2106, and Fighsare at https://doi.org/10.6084/m9.figshare.13473147. Phylogeny data were obtained from Dryad at https://doi.org/10.5061/dryad.tb03d03 and Nature at https://doi.org/10.1038/nature11631. Species range maps were obtained from the IUCN Red List at https://www.iucnredlist.org/resources/spatialdatadownload/.
