# Supplementary material for: Climate‐Driven Body Size Changes in Birds and Mammals Reveal Environmental Tolerance Limits
Source: Glob Chang Biol. 2025 May 9;31(5):e70241. doi: 10.1111/gcb.70241 (PMC12062781; doi:10.1111/gcb.70241)
Supplement: Supplementary file 1 — Figure S1. Spline correlograms testing for spatial autocorrelation of model residuals. No evidence of spatial autocorrelation is observed for (a) bird body mass model (Table S2), (b) mammal body mass model (Table S5), (c) bird body length model (Table S3), (d) mammal body length model (Table S6), (e) bird mass:length model (Table S4), (f) mammal mass:length model (Table S7). Distance reported in kilometers. Figure S2. Plot of model residuals against year for (a) Bird body mass, 846 out of 1000 samples showed no temporal autocorrelation (84.6%). (b) Mammal body mass, 951 out of 1000 samples showed no temporal autocorrelation (95.1%). (c) Bird body length, 955 out of 1000 samples showed no temporal autocorrelation (95.5%). (d) Mammal body length, 947 out of 1000 samples showed no temporal autocorrelation (94.7%). (e) Bird mass:length ratio, 865 out of 1000 samples showed no temporal autocorrelation (86.5%). (f) Mammal mass:length ratio, 949 out of 1000 samples showed no temporal autocorrelation (94.9%). Trend line fit using GAM (Residual ~ Year) to allow for potential nonlinear patterns (includes confidence intervals). Figure S3. Plot of model posterior predictive check for (a) Bird body mass (Table S2), (b) Mammal body mass (Table S5), (c) Bird body length (Table S3), (d) Mammal body length (Table S6), (e) Bird mass:length ratio (Table S4), (f) Mammal mass:length ratio (Table S7). 1000 simulated y values were generated from each model (y_sim) and compared to 1000 random sampled model response variable (y values). Figure S4. Taxonomic coverage results. Y axis represents the log10 transformed percentage each taxonomic order represents within the analyzed dataset. X axis represents the log10 transformed percentage of species each taxonomic order represents for the taxonomic classes Aves and Mammalia. The percentage of species distribution across the orders in our dataset are strongly correlated with the proportional representation of each Order within the taxonomic Clas [file GCB-31-e70241-s001.docx]

Supplementary Materials for

Climate change and land-use extent impact body size trends in birds and mammals

Matthew J. Watson*, Jeremy T. Kerr,

*Corresponding author. Email: [mwats041@uottawa.](mailto:mwats041@uottawa.)ca

**This PDF file includes:**

Supplementary Text

Supplementary Figures. 1 to 3

Supplementary Tables. 1 to 8

**Extended Data for this manuscript include the following and can be accessed through figshare:**

**Data_1_Bird_Mass (separate file):** Complete bird body mass data file used in analyses

**Data_2_Bird_Length (separate file):** Complete bird body length data file used in analyses

**Data_3_Bird_Size (separate file):** Complete bird body mass:length data file used in analyses

**Data_4_Mammal_Mass (separate file**): Complete mammal body mass data file used in analyses

**Data_5_Mammal_Length (separate file):** Complete mammal body length data file used in analyses

**Data_6_Mammal_Size (separate file):** Complete mammal body mass:length data file used in analyses

**Data_7_TPI_Limits (separate file):** Data file containing the thermal tolerance limits used for calculating Thermal Position Index.

**Data_8_Age_Classification (separate file):** Data file containing raw data age classification categories with corresponding manual age classification

**Data_9_Sex_Classification (separate file):** Data file containing raw data sex classification categories with corresponding manual sex classification

**mammalTree (separate file):** Phylogenetic tree used of mammal species for analyses

**FinalBirdTree_analysis (separate file):** Phylogenetic tree used of bird species for analyses

**Supplementary Text**

*Generating Thermal and Aridity Tolerance Limits*

Generating species-level environmental estimates can be accomplished using expert informed range maps or historical observations of species occurrences. Both methods exhibit biases when estimating species-environment relationships, but at large spatial scales both range maps and species observation data have been shown to generate similar species-level environmental estimates (Alhajeri & Fourcade, 2019). However, observation records tend to be dominated by larger, charismatic species, resulting in uneven representation across taxa, as well as displaying geographic biases towards easily accessible locations (Beck et al., 2014). Due to consistent species-level environmental estimates between both methods we decided to use range maps for deriving lower and upper thermal tolerance limits. This allowed us to avoid bias in geographic locations for less well sampled taxa within this study that could lead to biased estimates of thermal and aridity tolerance limits. We obtained expert-informed range maps from the International Union for the Conservation of Nature (IUCN, 2017a) (IUCN) for terrestrial mammals. IUCN data include a single map for each species of terrestrial mammal, which we used for extraction of regional temperature data for each species. For birds, we obtained range maps from BirdLife International (BirdLife International and Handbook of the Birds of the World, 2021).

To generate species-specific lower and upper thermal tolerance limits we obtained global minimum and maximum monthly temperature raster maps from the WorldClim database at a spatial resolution of ~21km2 for the years 1961 to 1975 (Fick & Hijmans, 2017). We used temperature data from 1961 to 1975, as this period serves as a baseline and is more representative of environmental conditions prior to rapid increases in temperature from anthropogenic climate change (Soroye et al., 2020). We calculated thermal limits for each month by extracting the minimum and maximum temperatures across each species’ range during the baseline period. We calculated the mean of all minimum and all maximum temperatures to obtain a lower and upper thermal limit value for each month, generating 12 upper and 12 lower thermal tolerance measurements for each species. Monthly limits were generated in order to account for potential variations in seasonal tolerances to environmental conditions due to behavioral adaptations or phenotypically plastic traits.

To determine upper and lower thermal tolerance limits for birds, we used BirdLife range maps, which are divided into resident maps (known to occur year-round), and seasonal maps (breeding season, non-breeding season, and passage). This allowed us to account for migratory movements when determining tolerance limits. For all species, we generated four range maps: Breeding (Resident and Breeding range maps), Non Breeding (Resident and Non Breeding range maps), Migration Non Breeding (Resident, Non Breeding, and Passage range maps), and Migration Breeding (Resident, Breeding, and Passage range maps) using ArcGIS Pro (Esri Inc., 2022). Migration Non Breeding range maps were generated to account for the early spring and late fall migration periods, when species will likely occur over much of their migratory range but are less likely to occur within their breeding range. Similarly, Migration Breeding range maps were generated to represent the late spring and early fall migration periods, when species likely still occur within much of their breeding range, but migration movements have begun to occur. Following the generation of seasonal range maps, we used biogeographic classifications from the IUCN Red List (IUCN, 2017b) to assign species to either Northern or Southern hemisphere distributions. This was done to account for different timings of breeding activity between Northern and Southern hemispheres due to differences in yearly seasonal periods. Species that had occurrences in the Palearctic or Nearctic were classified as Northern, while species that did not occur in these regions were classed as Southern.

We carried out all calculations for TPI limits as outlined for mammals, except that seasonal range maps were allocated to specific months based on species hemisphere classification. For northern species, we assigned Non Breeding range maps to the months December to February, Breeding range maps to the months May to September, Non Breeding Migration range maps to the months March and November, and Breeding Migration range maps to the months April and October. For southern species, we assigned Non Breeding range maps to the months June to August, Breeding range maps to the months November to March, Non Breeding Migration range maps to the months September and May, and Breeding Migration range maps to the months October and April. Finally, we combined all mammal, and bird species thermal limits into one comprehensive table (Extended Data 7).

*Taxonomic Distribution*

To evaluate if the species contained in our datasets were proportionally representative of the taxonomic classes of Aves and Mammalia, we first used ICUN Redlist taxonomic data to calculate the percentage of species contained within each order for its respective taxonomic classes. We then calculated the percentage of species in each order, for both Aves and Mammalia, that were contained within our datasets. After calculating the proportional distribution of species, we log10 transformed the percentage values to linearize the variables. We then ran a Pearson’s correlation between the two metrics of percent species composition by Order for those present in our datasets. We found that the percent distribution of species by taxonomic order was strongly correlated with the percent distribution of all species across orders for those present in our analyses (Figure S4). We acknowledge that some bird and mammal taxonomic orders do not have body size data and could not consequently be included in our analyses, but the taxonomic coverage of species in our dataset is proportionally representative at the order level for birds and mammals.

*Temporal Trends in Body Size*

We tested for temporal trends in body size metrics. We found independent relationships between body size metrics and year of observation for both bird and mammal species over the observation period of this study (1961-2018; Tables S2-S7), although the results varied between groups. Over time, mammals displayed an overall decline in body mass:length ratio (β = -0.0015, 95% CI = -0.0024 - -0.0007) (Figure 6b). This trend resulted from the combined impacts of increased body length (β = 0.084, 95% CI = 0.0022 - 0.0146) (Figure 5b) and stable body mass (β = -0.0014, 95% CI = -0.0078 - 0.0050) (Figure 3b) measurements over time. These patterns result in an overall decline in volume to surface area ratios in mammals, which is associated with greater thermoregulatory efficiency in warmer conditions (Gardner et al., 2011). This outcome is consistent with our predictions of body size responses to warming and drying trends associated with anthropogenic climate change.

Bird body mass increased over time (β = 0.0223, 95% CI = 0.0156 - 0.0289) (Figure 3a), independent from both land use and changing climatic conditions relative to species’ thermal and aridity limits. However, their body lengths decreased (β = -0.0248, 95% CI = -0.0477 - -0.0019) (Figure 5b). If increasing body sizes over time among bird species were associated with thermoregulatory capacity, then the ratio of mass:length temporally should also be increasing. However, there is no temporal trend for mass:length ratio in birds over time (β = -0.0015, 95% CI = -0.0163 - 0.0134) (Figure 6a). Additional data on bird body lengths could improve tests of this trend over time (Table S1).

Thermoregulatory efficiency among bird species is affected by the sizes of specific thermoregulatory structures, particularly beak surface area (Frӧhlich et al., 2023). Beaks are highly vascularized structures and modify bird thermoregulatory capacities (Greenberg et al., 2012b), which may complicate body size responses to temporal changes in TPI, API and land use. We speculate that increases in beak surface area over time could reduce the reliance of these species on simple body size metrics relative to their thermoregulatory efficiency as conditions warm. Future investigations into allometric effects of beak surface area on responses of birds to climate change driven warming and drying could offer novel insights into observed body size trends in birds.

**References**

Alhajeri, B. H., & Fourcade, Y. (2019). High correlation between species-level environmental data estimates extracted from IUCN expert range maps and from GBIF occurrence data. *Journal of Biogeography*, *46*(7), 1329–1341. <https://doi.org/10.1111/JBI.13619>

Beck, J., Böller, M., Erhardt, A., & Schwanghart, W. (2014). Spatial bias in the GBIF database and its effect on modeling species’ geographic distributions. *Ecological Informatics*, *19*, 10–15. https://doi.org/10.1016/J.ECOINF.2013.11.002

BirdLife International and Handbook of the Birds of the World. (2021). *Bird species distribution maps of the world. Version 2021.1.* Available at Http://Datazone.Birdlife.Org/Species/Requestdis.

Esri Inc. (2022). *ArcGIS Pro* (3.0). Esri Inc. https://www.esri.com/en-us/arcgis/products/arcgis-pro/overview.

Fick, S. E., & Hijmans, R. J. (2017). WorldClim 2: new 1-km spatial resolution climate surfaces for global land areas. *International Journal of Climatology*, *37*(12), 4302–4315. <https://doi.org/10.1002/JOC.5086>

Frӧhlich, A., Kotowska, D., Martyka, R., & Symonds, M. (2023) Allometry reveals trade-offs between Bergmann’s and Allen’s rules, and different avian adaptive strategies for thermoregulation. *Nat Comms* 14, 1101. <https://doi.org/10.1038/s41467-023-36676-w>

Gardner, J. L., Peters, A., Kearney, M. R., Joseph, L., & Heinsohn, R. (2011). Declining body size: A third universal response to warming? *Trends in Ecology and Evolution*, *26*(6), 285–291. https://doi.org/10.1016/j.tree.2011.03.005

Greenberg, R., Cadena, V., Danner, R.M. & Tattersall, G. (2012b) Heat loss may explain bill size differences between birds occupying different habitats. *PLoS ONE*, 7, e40933.

IUCN. (2017a). *The IUCN Red List of Threatened Species. version 3*. Https://Www.Iucnredlist.Org. Downloaded on 2022-09-12.

IUCN. (2017b, May). *IUCN Red List version 3*. Https://Www.Iucnredlist.Org. Downloaded on 2022-11-09.

Li, D., Dinnage, R., Nell, L. A., Helmus, M. R., & Ives, A. R. (2020). phyr: An r package for phylogenetic species-distribution modelling in ecological communities. *Methods in Ecology and Evolution*, *11*(11), 1455–1463. https://doi.org/10.1111/2041-210X.13471

**
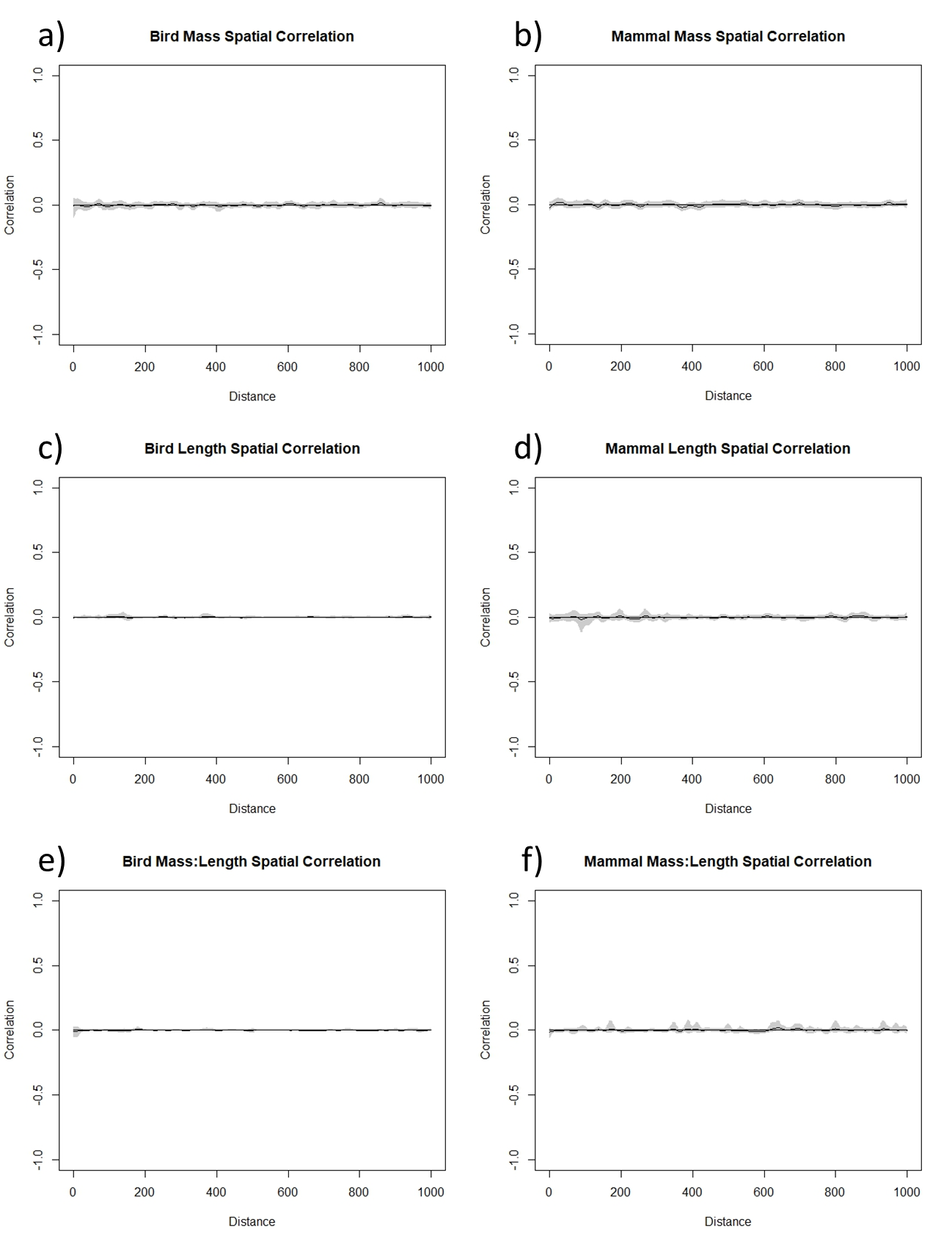
**

**Figure S1.** Spline correlograms testing for spatial autocorrelation of model residuals. No evidence of spatial autocorrelation is observed for **a)** bird body mass model (Supplementary Table 2), **b)** mammal body mass model (Supplementary Table 5), **c)** bird body length model (Supplementary Table 3), **d)** mammal body length model (Supplementary Table 6), **e**) bird mass:length model (Supplementary Table 4), **f**) mammal mass:length model (Supplementary Table 7),. Distance reported in kilometers.

**
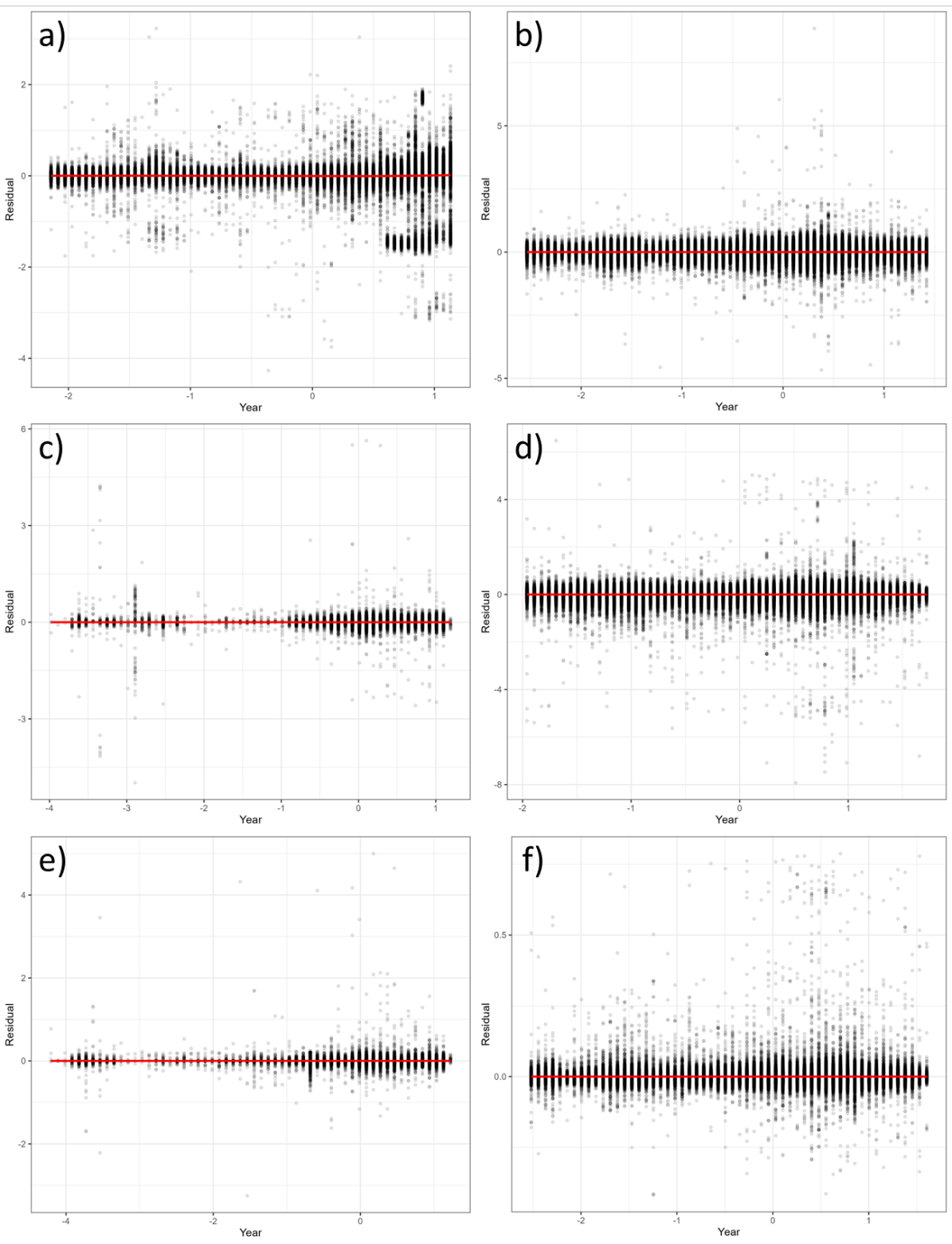
**

**Figure S2**. Plot of model residuals against year for **a)** Bird body mass, 846 out of 1000 samples showed no temporal autocorrelation (84.6%). **b**) Mammal body mass, 951 out of 1000 samples showed no temporal autocorrelation (95.1%). **c)** Bird body length, 955 out of 1000 samples showed no temporal autocorrelation (95.5%). **d)** Mammal body length, 947 out of 1000 samples showed no temporal autocorrelation (94.7%). e) Bird mass:length ratio, 865 out of 1000 samples showed no temporal autocorrelation (86.5%). f) Mammal mass:length ratio, 949 out of 1000 samples showed no temporal autocorrelation (94.9%). Trend line fit using GAM (Residual ~ Year) to allow for potential nonlinear patterns (includes confidence intervals).

**
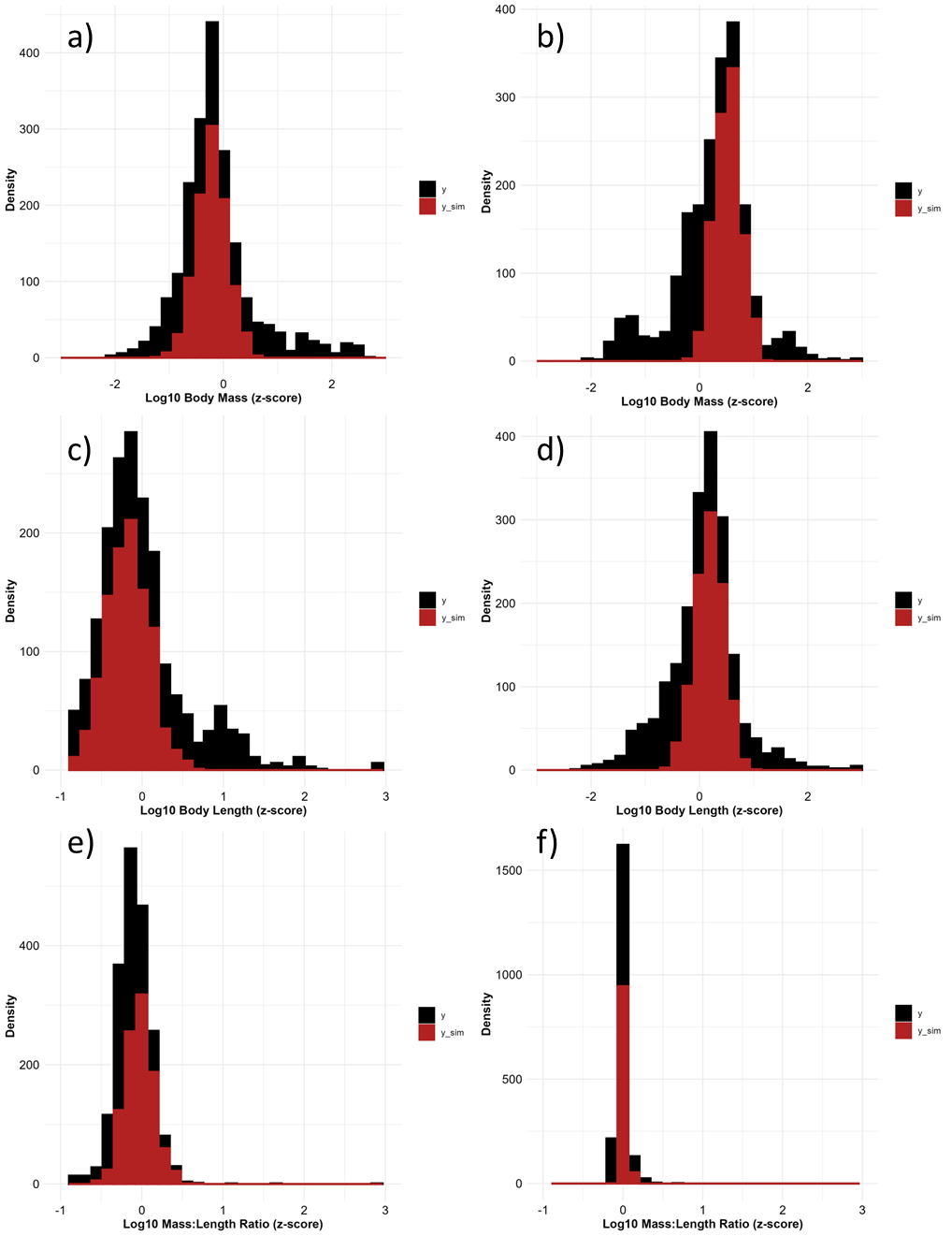
**

**Figure S3**. Plot of model posterior predictive check for **a**) Bird body mass (Supplementary Table 2) **b**) Mammal body mass (Supplementary Table 5), **c**) Bird body length (Supplementary Table 3), **d**) Mammal body length (Supplementary Table 6), **e**) Bird mass:length ratio (Supplementary Table 4), **f**) Mammal mass:length ratio (Supplementary Table 7). 1000 simulated y values were generated from each model (y_sim) and compared to 1000 random sampled model response variable (y values).


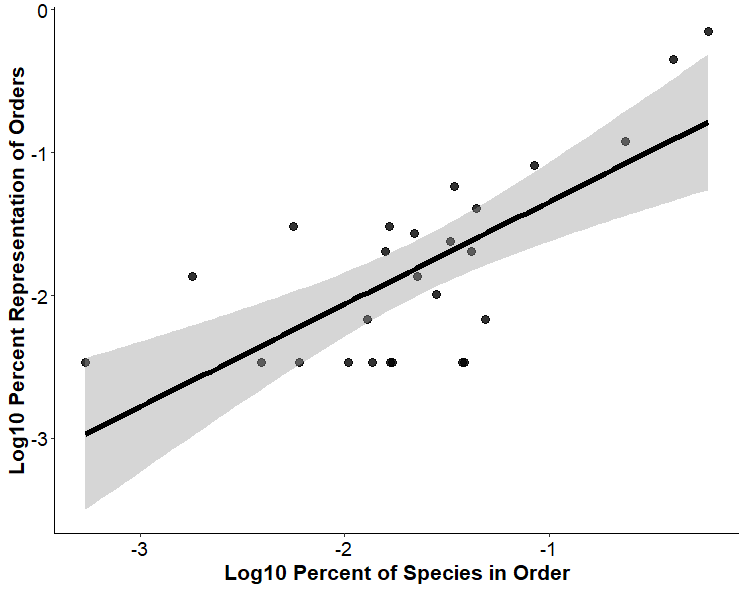


**Figure S4.** Taxonomic coverage results. Y axis represents the Log_10_ transformed percentage each taxonomic order represents within the analyzed dataset. X axis represents the Log_10_ transformed percentage of species each taxonomic order represents for the taxonomic classes Aves and Mammalia. The percentage of species distribution across the orders in our dataset are strongly correlated with the proportional representation of each Order within the taxonomic Classes of Aves and Mammalia (Pearson’s R = 0.705, 95% CI = 0.437 - 0.858, p = <0.001).

Table S1. Displays the number of observations, species, geographic sites, and biogeographic realms for each body size metric for both birds (Aves) and mammals (Mammalia).

| Taxonomic Class | Metric | Measurement | Observations (n) | Species (n) | Sites (n) | Biogeographic Realms (n) |
| --- | --- | --- | --- | --- | --- | --- |
| Aves | Body Mass | grams | 119183 | 370 | 15087 | 8 |
| Aves | Body Length | millimeters | 15562 | 63 | 1354 | 7 |
| Mammalia | Body Mass | grams | 183087 | 203 | 22826 | 8 |
| Mammalia | Body Length | millimeters | 239600 | 276 | 32097 | 8 |

Table S2. Reporting the Phylogenetic Generalized Linear Mixed Model results for bird Log_10_ body mass. The model was fit under a Bayesian framework (partial R^2^ = 0.930). Residual variance = 0.0774 (sd = 0.278). Beta estimates reported with 95% Credible Intervals and probability of direction (pd). Random effect variance reported with standard deviation and 95% Credible Intervals. A variable followed by |Binomial indicates a random slope effect by species, while |Binomial *P* indicates random slope effects with a control for phylogenetic correlation. Variance Inflation Factor (VIF) reported for each variable, showing no issues of variable collinearity.

| **Fixed Effects** | **Aves: Mass Response** | | | |  | |  |  |
| --- | --- | --- | --- | --- | --- | --- | --- | --- |
| **Predictor** | **Estimate** | | **95% Credible Intervals** | | | | **pd** | **VIF** |
| **(Intercept)** | **-0.2824** | | **-0.3039** | | **-0.2610** | | **1.00** |  |
| **TPI max** | **-0.0094** | | **-0.0146** | | **-0.0042** | | **0.99** | 1.015 |
| **API** | **0.0147** | | **0.0089** | | **0.0205** | | **1.00** | 1.239 |
| **ALU** | **0.0370** | | **0.0303** | | **0.0437** | | **1.00** | 1.059 |
| ULU | -0.0039 | | -0.0098 | | 0.0020 | | 0.91 | 1.084 |
| **Year** | **0.0223** | | **0.0156** | | **0.0289** | | **1.00** | 1.006 |
| **TPI max x API** | **0.0076** | | **0.0033** | | **0.0120** | | **1.00** | 1.230 |
| TPI max x ALU | -0.0006 | | -0.0044 | | 0.0032 | | 0.63 | 1.105 |
| TPI max x ULU | 0.0005 | | -0.0026 | | 0.0036 | | 0.60 | 1.126 |
|  |  | |  | |  | |  |  |
| **Random Effects** | **Variance** | **Standard Deviation** | | **95% Credible Intervals** | | | |  |
| TPI_Max\|Binomial | 0.0015 | 0.0387 | | 0.0011 | | 0.0025 | |  |
| TPI_Max\|Binomial *P* | 0.0001 | 0.0111 | | 0.0000 | | 0.0004 | |  |
| API\|Binomial | 0.0008 | 0.0290 | | 0.0005 | | 0.0014 | |  |
| API\|Binomial *P* | 0.0001 | 0.0085 | | 0.0000 | | 0.0002 | |  |
| ALU\|Binomial | 0.0004 | 0.0200 | | 0.0002 | | 0.0007 | |  |
| ALU\|Binomial *P* | 0.0002 | 0.0130 | | 0.0001 | | 0.0003 | |  |
| ULU\|Binomial | 0.0000 | 0.0044 | | 0.0000 | | 0.0000 | |  |
| ULU\|Binomial *P* | 0.0000 | 0.0039 | | 0.0000 | | 0.0000 | |  |
| Year\|Binomial | 0.0007 | 0.0273 | | 0.0005 | | 0.0013 | |  |
| Year\|Binomial *P* | 0.0001 | 0.0101 | | 0.0000 | | 0.0003 | |  |
| 1\|Binomial | 0.0037 | 0.0608 | | 0.0014 | | 0.0089 | |  |
| 1\|Binomial *P* | 0.1260 | 0.3549 | | 0.1108 | | 0.1486 | |  |
| 1\|Realm | 0.0146 | 0.1207 | | 0.0049 | | 0.0484 | |  |
| 1\|Site | 0.0920 | 0.3033 | | 0.0893 | | 0.0947 | |  |

Table S3. Reporting the Phylogenetic Generalized Linear Mixed Model results for bird Log_10_ body length. The model was fit under a Bayesian framework (partial R^2^ = 0.941). Residual variance = 0.0658 (sd = 0.2564). Beta estimates reported with 95% Credible Intervals and probability of direction (pd). Random effect variance reported with standard deviation and 95% Credible Intervals. A variable followed by |Binomial indicates a random slope effect by species, while |Binomial *P* indicates random slope effects with a control for phylogenetic correlation. Variance Inflation Factor (VIF) reported for each variable, showing no issues of variable collinearity.

| **Fixed Effects** | **Aves: Length Response** | | | |  | |  |  |
| --- | --- | --- | --- | --- | --- | --- | --- | --- |
| **Predictor** | **Estimate** | | **95% Credible Intervals** | | | | **pd** | **VIF** |
| **(Intercept)** | **-0.0970** | | **-0.2845** | | **-0.0221** | | **0.99** |  |
| **TPI max** | **-0.0181** | | **-0.0302** | | **-0.0062** | | **0.99** | 1.180 |
| API | -0.0077 | | -0.0323 | | 0.0167 | | 0.72 | 1.080 |
| ALU | -0.0004 | | -0.0651 | | 0.0645 | | 0.51 | 1.060 |
| ULU | -0.0037 | | -0.0459 | | 0.0385 | | 0.60 | 1.102 |
| **Year** | **-0.0248** | | **-0.0477** | | **-0.0019** | | **0.99** | 1.037 |
| TPI max x API | -0.0026 | | -0.0223 | | 0.0170 | | 0.60 | 1.096 |
| **TPI max x ALU** | **-0.0199** | | **-0.0344** | | **-0.0053** | | **0.99** | 1.188 |
| TPI max x ULU | -0.0024 | | -0.0138 | | 0.0091 | | 0.68 | 1.034 |
|  |  | |  | |  | |  |  |
| **Random Effects** | **Variance** | **Standard Deviation** | | **95% Credible Intervals** | | | |  |
| TPI_Max\|Binomial | 0.0001 | 0.0121 | | 0.0000 | | 0.0012 | |  |
| TPI_Max\|Binomial P | 0.0001 | 0.0081 | | 0.0000 | | 0.0025 | |  |
| API\|Binomial | 0.0000 | 0.0064 | | 0.0000 | | 0.0005 | |  |
| API\|Binomial P | 0.0031 | 0.0559 | | 0.0017 | | 0.0065 | |  |
| ALU\|Binomial | 0.0084 | 0.0919 | | 0.0049 | | 0.0155 | |  |
| ALU\|Binomial P | 0.0000 | 0.0065 | | 0.0000 | | 0.0005 | |  |
| ULU\|Binomial | 0.0000 | 0.0061 | | 0.0000 | | 0.0005 | |  |
| ULU\|Binomial P | 0.0014 | 0.0368 | | 0.0006 | | 0.0037 | |  |
| Year\|Binomial | 0.0059 | 0.0768 | | 0.0037 | | 0.0102 | |  |
| Year\|Binomial P | 0.0000 | 0.0068 | | 0.0000 | | 0.0008 | |  |
| 1\|Binomial | 0.0000 | 0.0064 | | 0.0000 | | 0.0006 | |  |
| 1\|Binomial P | 0.4110 | 0.6414 | | 0.2970 | | 0.6201 | |  |
| 1\|Realm | 0.0001 | 0.0105 | | 0.0000 | | 0.0035 | |  |
| 1\|Site | 1.3100 | 1.1438 | | 1.2100 | | 1.4194 | |  |

Table S4. Reporting the Phylogenetic Generalized Linear Mixed Model results for bird Log_10_ body mass:length ratio (∛Mass/Length). The model was fit under a Bayesian framework (partial R^2^ = 0.977). Residual variance = 0.0278 (sd = 0.1678). Beta estimates reported with 95% Credible Intervals and probability of direction (pd). Random effect variance reported with standard deviation and 95% Credible Intervals. A variable followed by |Binomial indicates a random slope effect by species, while |Binomial *P* indicates random slope effects with a control for phylogenetic correlation. Variance Inflation Factor (VIF) reported for each variable, showing no issues of variable collinearity.

| **Fixed Effects** | **Aves: Mass:Length Response** | |  |  |  |  |
| --- | --- | --- | --- | --- | --- | --- |
| **Predictor** | **Estimate** | **95% Credible Intervals** | | **pd** | **VIF** | |
| (Intercept) | 0.005303 | -0.03863 | 0.1023 | 0.55 |  | |
| **TPI max** | **-0.02015** | **-0.03305** | **-0.0073** | **0.99** | 1.005 | |
| **API** | **-0.01794** | **-0.03377** | **-0.0022** | **0.99** | 1.031 | |
| ALU | 0.016725 | -0.01499 | 0.0484 | 0.85 | 1.078 | |
| ULU | -0.0024 | -0.02715 | 0.0223 | 0.57 | 1.095 | |
| Year | -0.00146 | -0.01633 | 0.0134 | 0.57 | 1.012 | |
| TPI max x API | -0.00586 | -0.0132 | 0.0015 | 0.94 | 1.057 | |
| **TPI max x ALU** | **-0.0092** | **-0.01686** | **-0.0015** | **0.99** | 1.071 | |
| TPI max x ULU | 0.001818 | -0.00551 | 0.0091 | 0.68 | 1.055 | |
|  |  |  |  |  |  |  |
| **Random Effects** | **Variance** | **Standard Deviation** | **95% Credible Intervals** | |  |  |
| TPI_Max\|Binomial | 0.0013 | 0.0355 | 0.0003 | 0.0089 |  |  |
| TPI_Max\|Binomial *P* | 0.0097 | 0.0986 | 0.0061 | 0.0162 |  |  |
| API\|Binomial | 0.0156 | 0.1250 | 0.0109 | 0.0234 |  |  |
| API\|Binomial *P* | 0.0001 | 0.0045 | 0.0000 | 0.0001 |  |  |
| ALU\|Binomial | 0.0001 | 0.0052 | 0.0000 | 0.0001 |  |  |
| ALU\|Binomial *P* | 0.0001 | 0.0060 | 0.0000 | 0.0002 |  |  |
| ULU\|Binomial | 0.0001 | 0.0056 | 0.0000 | 0.0003 |  |  |
| ULU\|Binomial *P* | 0.0029 | 0.0535 | 0.0020 | 0.0041 |  |  |
| Year\|Binomial | 0.0170 | 0.1303 | 0.0134 | 0.0219 |  |  |
| Year\|Binomial *P* | 0.0001 | 0.0054 | 0.0000 | 0.0003 |  |  |
| 1\|Binomial | 0.0021 | 0.0460 | 0.0007 | 0.0085 |  |  |
| 1\|Binomial *P* | 0.0131 | 0.1146 | 0.0092 | 0.0202 |  |  |
| 1\|Realm | 0.0032 | 0.0566 | 0.0008 | 0.0313 |  |  |
| 1\|Site | 0.8211 | 0.9061 | 0.7734 | 0.8712 |  |  |

Table S5. Reporting the Phylogenetic Generalized Linear Mixed Model results for mammal Log_10_ body mass. The model was fit under a Bayesian framework (partial R^2^ = 0.949). Residual variance = 0.0555 (sd = 0.2355). Beta estimates reported with 95% Credible Intervals and probability of direction (pd). Random effect variance reported with standard deviation and 95% Credible Intervals. A variable followed by |Binomial indicates a random slope effect by species, while |Binomial *P* indicates random slope effects with a control for phylogenetic correlation. Variance Inflation Factor (VIF) reported for each variable, showing no issues of variable collinearity.

| **Fixed Effects** | **Mammalia: Mass Response** | | | |  | |  |  |
| --- | --- | --- | --- | --- | --- | --- | --- | --- |
| **Predictor** | **Estimate** | | **95% Credible Intervals** | | | | **pd** | **VIF** |
| **(Intercept)** | **0.5012** | | **0.4850** | | **0.5182** | | **1.00** |  |
| **TPI max** | **-0.0106** | | **-0.0164** | | **-0.0048** | | **0.99** | 1.009 |
| **API** | **0.0329** | | **0.0180** | | **0.0479** | | **1.00** | 1.006 |
| **ALU** | **0.0118** | | **0.0056** | | **0.0180** | | **1.00** | 1.016 |
| ULU | 0.0004 | | -0.0044 | | 0.0051 | | 0.56 | 1.055 |
| Year | -0.0014 | | -0.0078 | | 0.0050 | | 0.67 | 1.000 |
| **TPI max x API** | **0.0083** | | **0.0041** | | **0.0124** | | **1.00** | 1.004 |
| TPI max x ALU | -0.0015 | | -0.0047 | | 0.0017 | | 0.83 | 1.030 |
| TPI max x ULU | 0.0013 | | -0.0012 | | 0.0039 | | 0.67 | 1.070 |
|  |  | |  | |  | |  |  |
| **Random Effects** | **Variance** | **Standard Deviation** | | **95% Credible Intervals** | | | |  |
| TPI_Max\|Binomial | 0.0040 | 0.0631 | | 0.0028 | | 0.0063 | |  |
| TPI_Max\|Binomial *P* | 0.0004 | 0.0211 | | 0.0001 | | 0.0018 | |  |
| API\|Binomial | 0.0199 | 0.1409 | | 0.0147 | | 0.0286 | |  |
| API\|Binomial *P* | 0.0006 | 0.0236 | | 0.0002 | | 0.0023 | |  |
| ALU\|Binomial | 0.0010 | 0.0316 | | 0.0006 | | 0.0015 | |  |
| ALU\|Binomial *P* | 0.0001 | 0.0092 | | 0.0000 | | 0.0004 | |  |
| ULU\|Binomial | 0.0001 | 0.0117 | | 0.0001 | | 0.0006 | |  |
| ULU\|Binomial *P* | 0.0000 | 0.0057 | | 0.0000 | | 0.0001 | |  |
| Year\|Binomial | 0.0016 | 0.0394 | | 0.0010 | | 0.0024 | |  |
| Year\|Binomial *P* | 0.0003 | 0.0179 | | 0.0002 | | 0.0007 | |  |
| 1\|Binomial | 0.0043 | 0.0654 | | 0.0015 | | 0.0157 | |  |
| 1\|Binomial *P* | 0.2986 | 0.5464 | | 0.2467 | | 0.3846 | |  |
| 1\|Realm | 0.0070 | 0.0839 | | 0.0020 | | 0.0326 | |  |
| 1\|Site | 0.0614 | 0.2477 | | 0.0596 | | 0.0639 | |  |

Table S6. Reporting the Phylogenetic Generalized Linear Mixed Model results for mammal Log_10_ body length. The model was fit under a Bayesian framework (partial R^2^ = 0.936). Residual variance = 0.0700 (sd = 0.2646). Beta estimates reported with 95% Credible Intervals and probability of direction (pd). Random effect variance reported with standard deviation and 95% Credible Intervals. A variable followed by |Binomial indicates a random slope effect by species, while |Binomial *P* indicates random slope effects with a control for phylogenetic correlation. Variance Inflation Factor (VIF) reported for each variable, showing no issues of variable collinearity.

| **Fixed Effects** | **Mammalia: Length Response** | | | |  | |  |  |
| --- | --- | --- | --- | --- | --- | --- | --- | --- |
| **Predictor** | **Estimate** | | **95% Credible Intervals** | | | | **pd** | **VIF** |
| **(Intercept)** | **0.1781** | | **0.1620** | | **0.1944** | | **1.00** |  |
| **TPI max** | **-0.0052** | | **-0.0100** | | **-0.0005** | | **0.98** | 1.021 |
| **API** | **0.0169** | | **0.0040** | | **0.0298** | | **0.99** | 1.036 |
| ALU | 0.0019 | | -0.0040 | | 0.0078 | | 0.73 | 1.009 |
| **ULU** | **-0.0067** | | **-0.0114** | | **-0.0019** | | **0.99** | 1.022 |
| **Year** | **0.0084** | | **0.0022** | | **0.0146** | | **0.99** | 1.000 |
| **TPI max x API** | **0.0058** | | **0.0017** | | **0.0099** | | **0.99** | 1.028 |
| TPI max x ALU | 0.0013 | | -0.0018 | | 0.0043 | | 0.77 | 1.026 |
| TPI max x ULU | 0.0008 | | -0.0015 | | 0.0031 | | 0.74 | 1.042 |
|  |  | |  | |  | |  |  |
| **Random Effects** | **Variance** | **Standard Deviation** | | **95% Credible Intervals** | | | |  |
| TPI_Max\|Binomial | 0.0014 | 0.0372 | | 0.0010 | | 0.0022 | |  |
| TPI_Max\|Binomial *P* | 0.0001 | 0.0092 | | 0.0000 | | 0.0003 | |  |
| API\|Binomial | 0.0107 | 0.1035 | | 0.0080 | | 0.0160 | |  |
| API\|Binomial *P* | 0.0001 | 0.0106 | | 0.0000 | | 0.0004 | |  |
| ALU\|Binomial | 0.0015 | 0.0385 | | 0.0011 | | 0.0022 | |  |
| ALU\|Binomial *P* | 0.0001 | 0.0092 | | 0.0000 | | 0.0002 | |  |
| ULU\|Binomial | 0.0003 | 0.0178 | | 0.0002 | | 0.0005 | |  |
| ULU\|Binomial *P* | 0.0000 | 0.0064 | | 0.0000 | | 0.0001 | |  |
| Year\|Binomial | 0.0039 | 0.0623 | | 0.0032 | | 0.0047 | |  |
| Year\|Binomial *P* | 0.0000 | 0.0070 | | 0.0000 | | 0.0003 | |  |
| 1\|Binomial | 0.0346 | 0.1859 | | 0.0201 | | 0.0522 | |  |
| 1\|Binomial *P* | 0.1643 | 0.4054 | | 0.1398 | | 0.2108 | |  |
| 1\|Realm | 0.0099 | 0.0993 | | 0.0022 | | 0.0562 | |  |
| 1\|Site | 0.0896 | 0.2993 | | 0.0873 | | 0.0916 | |  |

Table S7. Reporting the Phylogenetic Generalized Linear Mixed Model results for mammal Log_10_ body mass:length ratio (∛Mass/Length). The model was fit under a Bayesian framework (partial R^2^ 0.868). Residual variance = 0.0010 (sd = 0.0313). Beta estimates reported with 95% Credible Intervals and probability of direction (pd). Random effect variance reported with standard deviation and 95% Credible Intervals. A variable followed by |Binomial indicates a random slope effect by species, while |Binomial *P* indicates random slope effects with a control for phylogenetic correlation. Variance Inflation Factor (VIF) reported for each variable, showing no issues of variable collinearity.

| **Fixed Effects** | **Mammalia: Mass:Length Response** | | | |  | |  |  |
| --- | --- | --- | --- | --- | --- | --- | --- | --- |
| **Predictor** | **Estimate** | | **95% Credible Intervals** | | | | **pd** | **VIF** |
| **(Intercept)** | **0.0218** | | **0.0197** | | **0.0240** | | **1.00** |  |
| **TPI max** | **-0.0012** | | **-0.0019** | | **-0.0004** | | **0.99** | 1.039 |
| API | -0.0012 | | -0.0028 | | 0.0005 | | 0.92 | 1.065 |
| **ALU** | **0.0015** | | **0.0006** | | **0.0023** | | **1.00** | 1.023 |
| ULU | 0.0003 | | -0.0005 | | 0.0011 | | 0.78 | 1.069 |
| **Year** | **-0.0015** | | **-0.0024** | | **-0.0007** | | **1.00** | 1.000 |
| TPI max x API | 0.0002 | | -0.0003 | | 0.0008 | | 0.80 | 1.047 |
| TPI max x ALU | -0.0002 | | -0.0007 | | 0.0002 | | 0.84 | 1.042 |
| TPI max x ULU | -0.0001 | | -0.0004 | | 0.0003 | | 0.64 | 1.088 |
|  |  | |  | |  | |  |  |
| **Random Effects** | **Variance** | **Standard Deviation** | | **95% Credible Intervals** | | | |  |
| TPI_Max\|Binomial | 0.00002 | 0.00396 | | 0.00001 | | 0.00003 | |  |
| TPI_Max\|Binomial *P* | 0.00001 | 0.00379 | | 0.00001 | | 0.00002 | |  |
| API\|Binomial | 0.00002 | 0.00480 | | 0.00001 | | 0.00004 | |  |
| API\|Binomial *P* | 0.00002 | 0.00429 | | 0.00001 | | 0.00003 | |  |
| ALU\|Binomial | 0.00002 | 0.00436 | | 0.00001 | | 0.00004 | |  |
| ALU\|Binomial *P* | 0.00001 | 0.00259 | | 0.00000 | | 0.00001 | |  |
| ULU\|Binomial | 0.00001 | 0.00289 | | 0.00000 | | 0.00001 | |  |
| ULU\|Binomial *P* | 0.00001 | 0.00247 | | 0.00000 | | 0.00001 | |  |
| Year\|Binomial | 0.00001 | 0.00369 | | 0.00001 | | 0.00003 | |  |
| Year\|Binomial *P* | 0.00002 | 0.00489 | | 0.00002 | | 0.00004 | |  |
| 1\|Binomial | 0.00007 | 0.00850 | | 0.00002 | | 0.00018 | |  |
| 1\|Binomial *P* | 0.00143 | 0.03784 | | 0.00109 | | 0.00173 | |  |
| 1\|Realm | 0.00000 | 0.00059 | | 0.00000 | | 0.00067 | |  |
| 1\|Site | 0.00095 | 0.03078 | | 0.00092 | | 0.00098 | |  |

Table S8. Comparison of number of observations from original datasets to filtered datasets. Filtered datasets contain all observations before filtering species based on the requirement of 100 observations for inclusion.

| Dataset | Taxonomic Class | Original Observation count (n) | Filtered Observation count (n) | Original Species Count (n) | Filtered Species Count (n) |
| --- | --- | --- | --- | --- | --- |
| *ATLANTIC: Data Papers* | Aves | 72,483 | 38,264 | 1,314 | 140 |
| *ATLANTIC: Data Papers* | Mammalia | 39,850 | 0 | 279 | 0 |
| *National Ecological Observatory* | Mammalia | 878,302 | 24,178 | 147 | 36 |
| *Ocampo et al* | Aves | 41,722 | 21,342 | 639 | 172 |
| *Ocampo et al* | Mammalia | 7,331 | 273 | 269 | 2 |
| *VertNet* | Aves | 5,934,806 | 60,621 | 20,319 | 363 |
| *VertNet* | Mammalia | 4,345,250 | 235,994 | 12,965 | 274 |

Table S9. Reporting the Phylogenetic Generalized Linear Mixed Model results for bird Log_10_ body mass, length, and mass:length ratio (∛Mass/Length) with the year variable term removed. These models highlight that our models (Supplementary Tables S2-S4) are robust to the inclusion of year as a covariate term. The models were fit under a Bayesian with Beta estimates reported with 95% Credible Intervals. All variables and random effects are conserved between the main models (Supplementary Tables S2-S4) and the models reported here. A variable followed by |Binomial indicates a random slope effect by species, while |Binomial *P* indicates random slope effects with a control for phylogenetic correlation.

|  | **Aves** | | | | | |
| --- | --- | --- | --- | --- | --- | --- |
|  | **Body Mass** | | **Body Length** | | **Mass:Length** | |
|  | *Est* | *95% CI* | *Est* | *95% CI* | *Est* | *95% CI* |
| Intercept | **-0.2887** | **-0.3111 - -0.2663** | **-0.0888** | **-0.1757 - -0.021** | **-0.0122** | **-0.0380 - 0.0145** |
| TPI | **-0.0091** | **-0.0144 - -0.0038** | **-0.0216** | **-0.0349 - -0.0089** | **-0.0174** | **-0.0294 - -0.0054** |
| API | **0.0181** | **0.0115 - 0.0247** | -0.0093 | -0.0333 - 0.0146 | **-0.0176** | **-0.0329 - -0.0023** |
| ALU | **0.0398** | **0.0329 - 0.0467** | -0.0047 | -0.0686 - 0.0593 | 0.0104 | -0.0238 - 0.0445 |
| ULU | -0.0044 | -0.0113 - 0.0024 | -0.0128 | -0.0547 - 0.0292 | -0.0040 | -0.0281 - 0.0201 |
| TPI x API | **0.0099** | **0.0049 - 0.0149** | 0.0014 | -0.0179 - 0.0207 | -0.0052 | -0.0125 - 0.0021 |
| TPI x ALU | -0.0003 | -0.0042 - 0.0036 | -0.0144 | -0.0295 - 0.0008 | **-0.0137** | **-0.0225 - -0.0047** |
| TPI x ULU | 0.0001 | -0.0034- 0.0034 | -0.0040 | -0.0159 - 0.008 | 0.0021 | -0.0052 - 0.0094 |

Table S10. Reporting the Phylogenetic Generalized Linear Mixed Model results for mammal Log_10_ body mass, length, and mass:length ratio (∛Mass/Length) with the year variable term removed. These models highlight that our models (Supplementary Tables S5-S7) are robust to the inclusion of year as a covariate term. The models were fit under a Bayesian with Beta estimates reported with 95% Credible Intervals. All variables and random effects are conserved between the main models (Supplementary Tables S5-S7) and the models reported here. A variable followed by |Binomial indicates a random slope effect by species, while |Binomial *P* indicates random slope effects with a control for phylogenetic correlation.

|  | **Mammalia** | | | | | |
| --- | --- | --- | --- | --- | --- | --- |
|  | **Body Mass** | | **Body Length** | | **Mass:Length** | |
|  | *Est* | *95% CI* | *Est* | *95% CI* | *Est* | *95% CI* |
| Intercept | **0.5007** | **0.4851 - 0.5162** | **0.1869** | **0.1708 - 0.203** | **0.0206** | **0.0185 - 0.0227** |
| TPI | **-0.0110** | **-0.0167 - -0.0053** | -0.0041 | -0.0090 - 0.0008 | **-0.0012** | **-0.0019 - -0.0004** |
| API | **0.0330** | **0.0181 - 0.048** | **0.0193** | **0.0055 - 0.033** | -0.0017 | -0.0034 - 0.0001 |
| ALU | **0.0121** | **0.0060 - 0.0182** | 0.0010 | -0.0050 - 0.007 | **0.0013** | **0.0004 - 0.0021** |
| ULU | 0.0001 | -0.0043 - 0.0045 | **-0.0065** | **-0.0117 - -0.0013** | 0.0002 | -0.0005 - 0.0011 |
| TPI x API | **0.0087** | **0.0045 - 0.0128** | **0.0057** | **0.0016 - 0.0098** | 0.0003 | -0.0002 - 0.0009 |
| TPI x ALU | -0.0015 | -0.0046 - 0.0017 | 0.0014 | -0.0016 - 0.0045 | -0.0002 | -0.0006 - 0.0002 |
| TPI x ULU | 0.0016 | -0.0008 - 0.0042 | 0.0007 | -0.0016 - 0.003 | 0.0000 | -0.0003 - 0.0004 |
